# Association between the Maternal Mediterranean Diet and Perinatal Outcomes: A Systematic Review and Meta-Analysis

**DOI:** 10.1016/j.advnut.2023.100159

**Published:** 2023-12-01

**Authors:** Jirong Xu, Haixia Wang, Jingfeng Bian, Ming Xu, Nan Jiang, Wei Luo, Ping Zu, Wanjun Yin, Peng Zhu

**Affiliations:** 1 Department of Maternal, Child & Adolescent Health, School of Public Health, Anhui Medical University, Hefei, China; 2 MOE Key Laboratory of Population Health Across Life Cycle, Hefei, China; 3 NHC Key Laboratory of Study on Abnormal Gametes and Reproductive Tract, Anhui Medical University, Hefei, China; 4 Anhui Provincial Key Laboratory of Environment and Population health across the Life Course, Anhui Medical University, Hefei, China; 5 Center for Big Data and Population Health of IHM, School of Public Health, Anhui Medical University, Hefei, China

**Keywords:** Mediterranean diet, dietary pattern, pregnancy, perinatal outcomes, fetal outcomes, maternal outcomes, neonatal outcomes, meta-analysis

## Abstract

The Mediterranean diet is a global, well-known healthy dietary pattern. This review aims to synthesize the existing evidence on the relationship between the maternal Mediterranean diet during pregnancy and perinatal outcomes, including randomized controlled trials (RCTs) and cohort studies. PubMed, Web of Science, and the Cochrane Library were searched from inception to 10 March, 2023, supplemented by manual screening. A random-effect model was used to estimate pooled sizes with 95% confidence intervals (CIs) for specific outcomes of interest. Data from 5 RCTs and 18 cohort studies with 107,355 pregnant participants were synthesized. In RCTs, it was observed that the maternal Mediterranean diet significantly reduced the incidence of gestational diabetes mellitus [odds ratio (OR), 0.56; 95% CI: 0.34, 0.93], as well as small for gestational age (0.55; 95% CI: 0.35, 0.88). In cohort studies, the highest adherence score to the maternal Mediterranean diet was inversely associated with a lower risk of various adverse pregnancy outcomes, including gestational diabetes mellitus (OR, 0.82; 95% CI: 0.67, 1.00), pregnancy-induced hypertension (0.73; 95% CI: 0.60, 0.89), pre-eclampsia (0.77; 95% CI: 0.64, 0.93), preterm delivery (0.67; 95% CI: 0.49, 0.91), low birth weight (0.70; 95% CI: 0.64, 0.78), intrauterine growth restriction (0.46; 95% CI: 0.23, 0.91), and increased gestational age at delivery (weighted mean difference, 0.11 wk; 95% CI: 0.03, 0.20). Meta-regression analyses did not identify the adjustment for confounders and geographical location as predictive factors for heterogeneity. The results suggest that adherence to the Mediterranean diet during pregnancy appears to be beneficial for perinatal outcomes. Future, larger, and higher-quality RCTs and cohort studies are warranted to confirm the present findings.

**PROSPERO registration no.**: CRD42023406317.

**Statement of significance**

The Mediterranean diet during pregnancy may provide perinatal benefits but no systematic review to date has examined its effect on a multitude of outcomes related to perinatal health. Therefore, well-designed and -conducted prospective cohort studies and randomized controlled trials are the most robust methodological approach to examine the relation between diet and health.

## Introduction

Maternal malnutrition during pregnancy is a potentially modifiable risk factor that can impact the health of both the mother and the newborn [[1], [2], [3]]. It plays a crucial role in supporting the growth of organs, muscles, and bones, as well as maintaining physiological and metabolic health. Therefore, improving dietary patterns and nutritional status throughout pregnancy can significantly contribute to preventing conditions such as gestational diabetes, preterm delivery, and their associated adverse effects on perinatal health [[4], [5], [6]]. This perspective is consistent with the current focus on the first 1000 d of life, from conception to 24 mo of age, as a critical window of opportunity to promote intergenerational health.

The Mediterranean diet (MD) is a well-known healthy and balanced dietary pattern [[7], [8], [9]]. The traditional MD is characterized by an abundant intake of vegetables, fruit as dessert, whole grains, nuts, legumes, fish, and the use of olive oil as a source of fat. This diet's components, primarily known for their anti-inflammatory and antioxidant properties, are thought to have favorable effects on various aspects of health, including cardiovascular disease (CVD) [10,11], metabolic health [12], autoimmune conditions [13], mental well-being [14], reduced cancer risk [15], lower overall mortality [11,16,17], and the promotion of healthy aging [18]. Given that pregnancy underlines the balanced and nutritious diet [19], there is a need to investigate the potential impact of the MD on maternal and neonatal health during this critical period.

Several studies have found that the MD appears to be associated with a lower risk of diabetes [20], gestational hypertension [21], low birth weight neonates [22], and favorable metabolic profiles in the offspring [7,23]; however, the results are inconsistent. For instance, although the association between improved metabolic health and the MD is well established in non-pregnant populations [10,18], our understanding of this relationship among pregnant females remains limited. Furthermore, previous systematic reviews have explored the influence of Mediterranean dietary patterns during pregnancy on perinatal outcomes [24,25] and children’s health [26,27]. However, it is worth noting that Amati et al. [24] did not employ meta-analytic techniques to synthesize the results, and Zhang et al. [25] included a limited number of randomized controlled trials (RCTs) with outcomes pooled. In general, the epidemiological evidence on the relationship between the MD and perinatal outcomes remains inconclusive, indicating the need for a comprehensive analysis to gain a deeper understanding phenomenon and interpret available data. It is worth mentioning that in most of these syntheses, prospective, and cross-sectional associations were combined with interventional ones, making them more susceptible to recall bias and reverse causation. To provide more clarity on this relationship, we conducted a systematic review and meta-analysis focusing on RCTs and cohort studies to quantify the association between maternal adherence to the MD and perinatal outcomes. This approach aimed to address the inconsistent reporting of results in existing studies.

## Methods

We followed the Meta-analysis of Observational Studies in Epidemiology and PRISMA guidelines for reporting this systematic review [28,29]. The study protocol was established and registered on PROSPERO, an international prospective register of systematic reviews [CRD42023406317].

### Searching strategy

A comprehensive literature search was conducted in 3 electronic databases, including PubMed, Web of Science, and the Cochrane Library, up to 10 March, 2023. Briefly, the following Medical Subject Headings (MeSH) and non-MeSH terms were used: “Mediterranean diet,” “dietary pattern,” “neonatal∗,” “pregnan∗,” “prenatal∗,” “maternal,” “gestation∗,” “antepartum∗,” “deliver∗,” and “periconception∗.” Additional details regarding the search strategy can be found in Supplemental Table 1. We included a wide of studies, including cohort studies and RCTs, with no restrictions on data, or original publications. Furthermore, we performed a manual search of the bibliographies of relevant reviews and meta-analyses to ensure a comprehensive data set. Two independent investigators (J-RX and J-FB) selected the articles according to the prespecified inclusion and exclusion criteria, and any disagreements were resolved by the principal investigator (MX).

### Study selection

Studies were included if they met the following criteria: *1*) exposure: adherence to the MD during pregnancy; *2*) outcome: at least 1 perinatal outcome or birth variable in the mother and the infant; *3*) population: generally healthy pregnant females without a history of diabetes, hypertension, CVD, chronic kidney disease, and metabolic/genetic syndromes, aged 18 y and older; *4*) design: RCTs and cohort studies; *5*) original data: reported estimates of relative risks (RRs), odds ratios (ORs), or mean differences with corresponding 95% confidence intervals (CIs) for the association of adherence to the MD with perinatal outcomes, or sufficient data to estimate both; and *6*) when multiple reports from the same cohort study were presented, only the most recent reports with the largest number of participants for identical outcomes were included. Exclusion criteria were non-original articles (reviews, commentaries, editorials, or letters).

### Data extraction and quality assessment

Data from the included studies were extracted independently by 2 investigators (J-RX and J-FB). The following information was extracted from each eligible article: first author, publication year, country, study design, sample size, race/ethnicity, age, dietary assessment, the MD assessment, highest compared with lowest adherence to MD, outcomes, adjustments for confounders (if any), and crude and adjusted estimates. If a study provided multiple estimates, we used those from the most complex model (that is, the one that included the largest number of confounders). For studies that did not provide adjusted estimates, we calculated ORs and 95% CIs using the available extracted data.

We assessed the design, execution, and reporting of each RCT included in this meta-analysis according to the Cochrane risk of bias tool [30] The Newcastle-Ottawa Scale (NOS) was used to assess the quality of cohort studies, with scores ranging from 0 to 9 [31]. Scores of 0–3, 4–6, and 7–9 indicate low, medium, and high quality, respectively [32]. Disagreements were resolved by discussion and by the opinion of a third author (MX). The results of the quality assessment of the studies included in the meta-analysis are shown in Supplemental Figure 1 and Supplemental Table 2.

### Outcomes

We included studies involving pregnant females from the general population that investigated the association between adherence to the MD and risk or odds of perinatal outcomes. In our analysis, we focused on maternal outcomes, which included gestational diabetes mellitus (GDM), pregnancy-induced hypertension, pre-eclampsia, preterm birth, and cesarean section. In addition, we examined fetal or neonatal outcomes, which included small for gestational age (SGA), large for gestational age, low birth weight, intrauterine growth restriction, and admission to the neonatal intensive care unit admission. We also included continuous outcomes for perinatal assessments, specifically gestational age at delivery and birth weight. The data concerning these perinatal outcomes were derived from a combination of self-reports, clinician notes, and hospital electronic records obtained after the delivery process in all the studies included in our analysis.

### Statistical analysis

We conducted meta-analyses when at least 3 studies provided data for a particular outcome, categorized the data into 3 or 4 levels of adherence, and compared the results between the lowest and highest adherence levels. In our analysis, we quantified the study outcomes using ORs and weighted mean differences (WMD) with 95% CIs. We used the following formula to convert RR to ORs: OR = RR × (1 − *P*) / (1 − (*P* × RR)), where *P* is the typical event rate without treatment [33]. All our meta-analyses were performed using random-effects models. Sensitivity analysis was performed to estimate the robustness of the results by omitting one study in turn to determine if an individual study or a group study had considerable influence on our results. We used the *I*^2^ statistic to assess the statistical heterogeneity within studies. An *I*^2^ value determined the variability of results between different studies as either low (25%), moderate (50%), or high (75%) [34]. To identify the source of heterogeneity among the studies, we performed subgroup and meta-regression analyses using the following factors: confounders (that is, adjusted, and unadjusted), and geographical location (that is, Mediterranean, and non-Mediterranean countries). Nevertheless, when the number of studies reporting on a specific outcome was limited, we did not perform a meta-analysis and stratification. Because all perinatal outcomes had a limited number of studies (<10), publication bias was not assessed. All statistical analyses were performed using Stata 17.0 (Stata 17 MP). A *P* value < 0.05 in 2-tailed tests was considered statistically significant.

## Results

### Literature search

**FIGURE 1:**
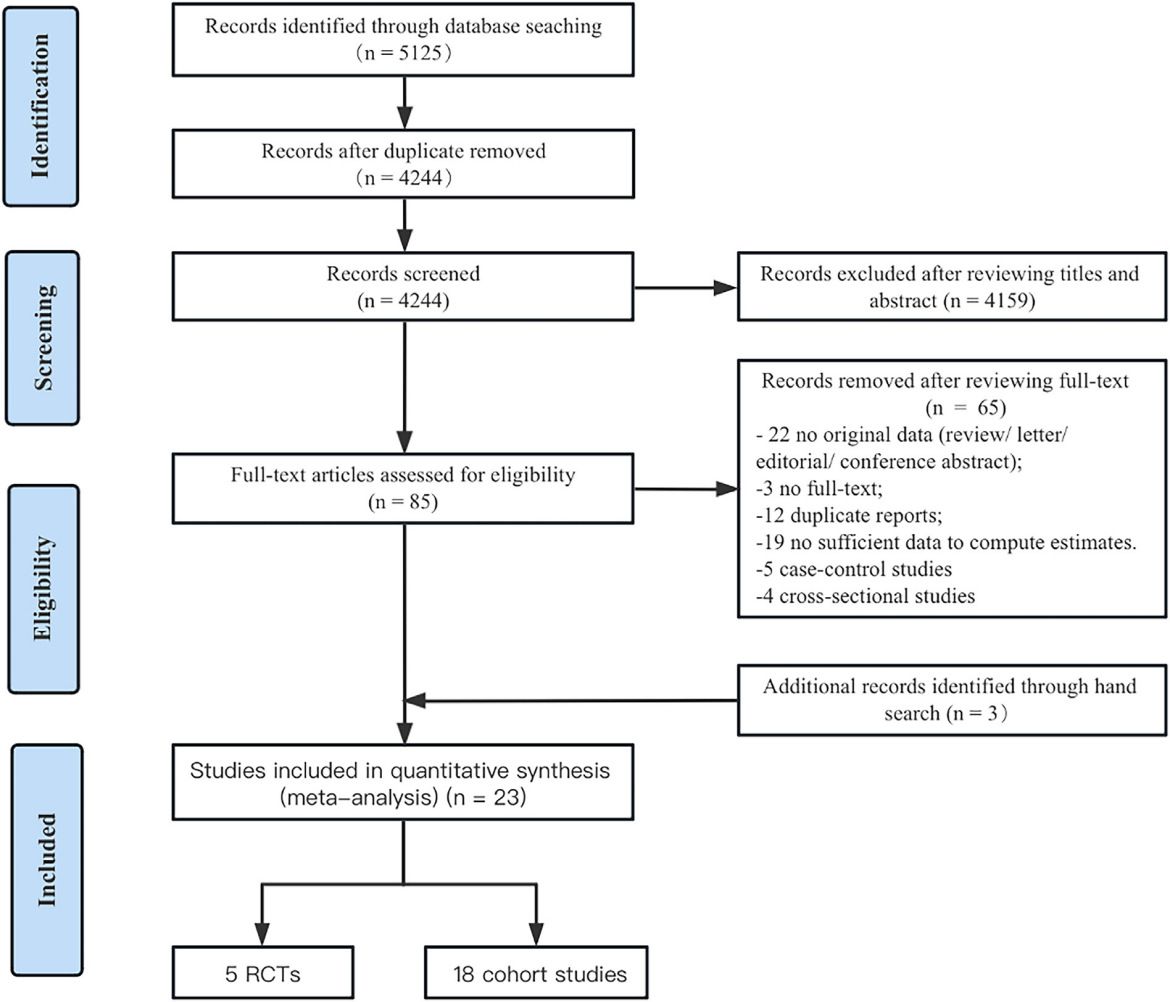
Flowchart of the number of studies identified and included in the systematic review and meta-analysis. RCT, randomized controlled trial.

The flowchart for the literature search is presented in Figure 1. Our initial search identified 5125 articles from 3 databases. Of these, 5040 articles were excluded based on the basis of duplicates, titles, and abstracts. By reviewing the full text, we removed 65 articles (Supplemental Table 3). In addition, we included 3 articles after hand-searching. A total of 23 articles, including 5 RCTs, and 18 cohort studies, met the eligibility criteria for this quantitative synthesis.

### Study characteristics

The basic information of the eligible RCTs and cohort studies is listed in Supplemental Tables 4 and 5, respectively. In summary, 5 RCTs [[35], [36], [37], [38], [39]] and 18 cohort studies [21,[40], [41], [42], [43], [44], [45], [46], [47], [48], [49], [50], [51], [52], [53], [54], [55], [56]] were included in the meta-analysis. The analysis includes a total of 107,355 subjects from 10 different countries, consisting of the United Kingdom [35,56], Spain [36,37,39,51,53], China [38], United States [41,44,45,49,50,54], Greece [46], Norway [42], Denmark [43], Australia [21,52], the Netherlands [47,4], or in multiple centers (that is, Spain and Greece) [40], and 10 Mediterranean countries [55]). The number of participants varied between 82 and 35,530, and the publication years ranged from 2008 to 2022. Almost all the studies used the food frequency questionnaire (FFQ) to assess dietary intake.

### Risk of bias

The individual quality assessment of the 5 RCTs showed a low risk of bias for most of the domains assessed. However, because of the inherent nature of dietary studies, the blinding of participants and personnel was identified as a high risk of bias (see Supplemental Figure 1). According to the criteria of the NOS (Supplemental Table 2), 3 cohort studies [41,45,46] were assessed as being of medium quality, whereas 15 cohort studies [21,40,[42], [43], [44],[47], [48], [49], [50], [51], [52], [53], [54], [55], [56]] were rated as high quality.

### Associations between the MD and perinatal outcomes

**FIGURE 2:**
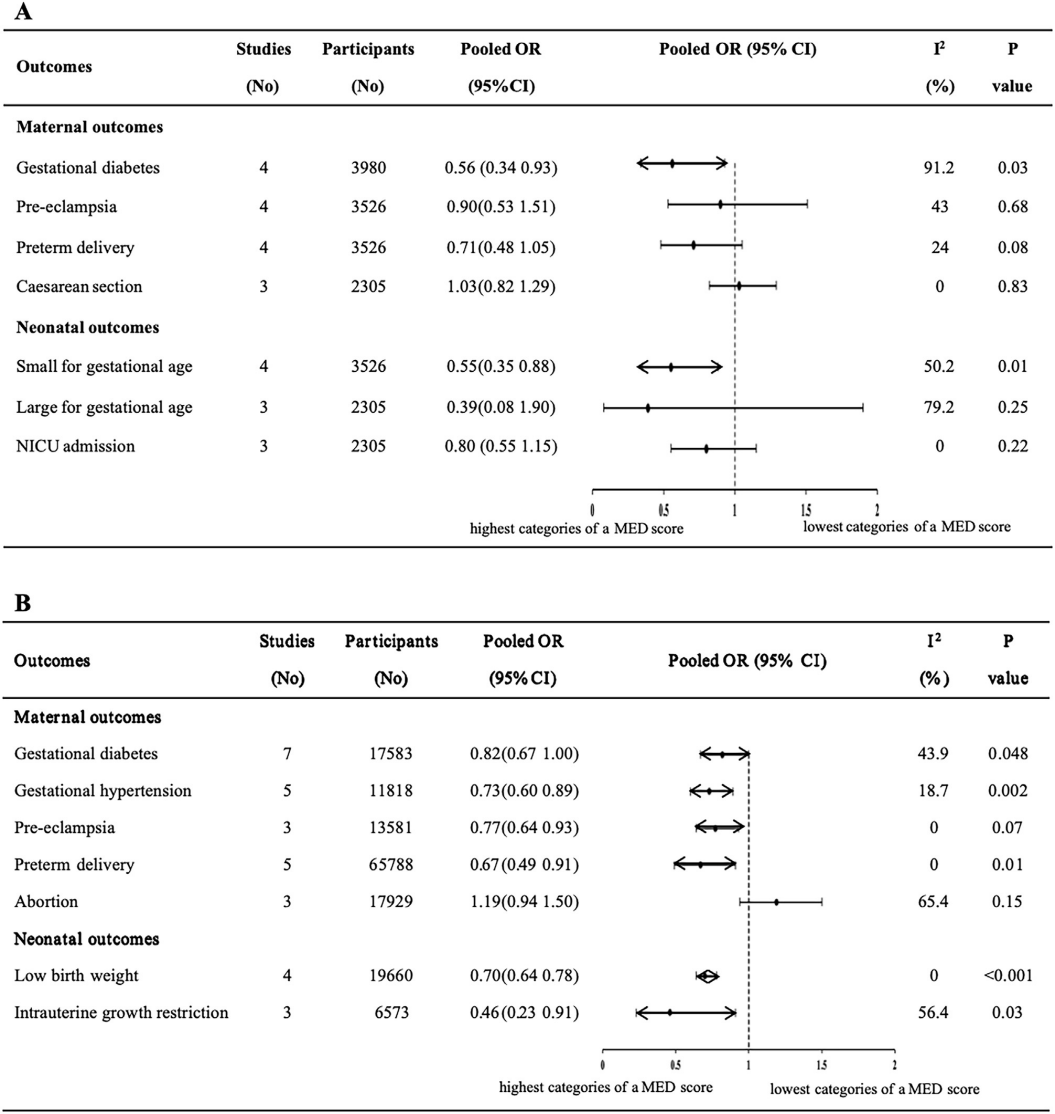
Findings of meta-analysis of the association between maternal MD and risk of adverse perinatal outcomes. (A) The included RCTs and (B) the included cohort studies. The diamond denotes the summary risk estimate, and horizontal lines represent the 95% CI. Abbreviations: CI, confidence interval; MD, Mediterranean diet; OR, odds ratio.

According to the 5 included RCTs, the association between the maternal MD and perinatal outcomes is shown in Figure 2A, Table 1, and Supplemental Figure 2. Compared with the control group of pregnant females, the MD was associated with a significantly lower incidence of GDM (OR, 0.56; 95% CI: 0.34, 0.93; *I*^2^ = 91.2%; *n* = 4 studies) and SGA (OR, 0.55; 95% CI: 0.35, 0.88; *I*^2^ = 50.2%; *n* = 4) (Figure 2A). Stratification and sensitivity analyses were not performed because of the small number of included studies.

**TABLE 1:** Pooled WMD from the primary meta-analysis of the effects of the Mediterranean diet during pregnancy on birth weight and gestational age at delivery

Outcomes	Studies (*N*)	Participants (*N*)	Pooled WMD (95% CI)	*I*^2^ (%)	*P* value
RCTs					
Birth weight (g)	3	2305	52.67 (−9.39, 114.73)	37.2	0.1
Cohort studies					
Gestational age at delivery (wk)	5	13,899	0.11 (0.03, 0.20)	27.5	0.007
Birth weight (g)	6	34,058	23.06 (−29.82, 75.94)	68.5	0.39

Abbreviations: CI, confidence interval; RCT, randomized controlled trial; WMD, weighted mean difference.

In 18 cohort studies, the maternal MD demonstrated significant benefits in 6 of 17 perinatal outcomes of interest (Figure 2B, Table 1, and Supplemental Figure 3). When comparing the highest and lowest MD scores, the MD was associated with reduced odds of GDM (OR, 0.82; 95% CI: 0.67, 1.00; *I*^2^ = 43.9%; *n* = 7), pregnancy-induced hypertension (OR, 0.73; 95% CI: 0.60, 0.89; *I*^2^ = 18.7%; *n* = 5), pre-eclampsia (OR, 0.77; 95% CI: 0.64, 0.93; *I*^2^ = 0.0%; *n* = 3), preterm delivery (OR, 0.67; 95% CI: 0.49, 0.91; *I*^2^ = 0.0%; *n* = 5), low birth weight (OR, 0.70; 95% CI: 0.64, 0.78; *I*^2^ = 0.0%; *n* = 4), intrauterine growth restriction (OR, 0.46; 95% CI: 0.23, 0.91; *I*^2^ = 56.4%; *n* = 3), and an increase in gestational age at delivery (WMD, 0.11 wk; 95% CI: 0.03, 0.20; *I*^2^ = 27.5%; *n* = 5).

### Subgroup, meta-regression, and sensitivity analyses in cohort studies

The meta-analysis concerning the relationship between the maternal MD and perinatal outcomes revealed varying levels of heterogeneity. Moderate heterogeneity was observed for GDM, abortion, intrauterine growth restriction, gestational age at delivery, and birth weight (*I*^2^ = 43.9%, 65.4%, 56.4%, 27.5%, and 68.5%, respectively). However, the levels of heterogeneity for pregnancy-induced hypertension, pre-eclampsia, preterm delivery, and low birth weight were either low or non-existent (*I*^2^ = 18.7%, 0%, 0%, and 0%, respectively). Furthermore, sensitivity analyses examining the relationship between the maternal MD and GDM, pregnancy-induced hypertension, preterm delivery, gestational age at delivery, and birth weight did not substantially alter the overall pooled results (Supplemental Figure 6).

To assess the robustness of risk estimate, we conducted stratified analyses based on the adjustment of confounders and geographical location (Table 2 and Supplemental Figures 4 and 5). Regarding GDM, the stratification generally did not significantly affect the pooled estimate of the association, with 1 exception: in the 2 studies that adjusted for confounders, the pooled OR decreased to 0.72 (95% CI: 0.58, 0.90). For pregnancy-induced hypertension, both the adjusted and non-Mediterranean country subgroups showed a statistically significant association between the maternal MD and the incidence of pregnancy-induced hypertension (OR, 0.73; 95% CI: 0.64, 0.83; *n* = 3), whereas this association was not observed in the unadjusted or Mediterranean country groups. In the case of preterm delivery, a significant correlation between the maternal MD and preterm delivery incidence was found in the adjusted group (OR, 0.60; 95% CI: 0.40, 0.89; *n* = 3). However, the remaining analyses within the unadjusted subgroup showed significant association. For birth weight, we observed a significant WMD in the non-Mediterranean country subgroup (WMD, 50.73 g; 95% CI: 14.03, 87.44; *n* = 3), whereas no significant association was found in the Mediterranean country subgroup. Although meta-regression analyses did not reveal any predictive power for heterogeneity in terms of adjusting for confounders and geographical location, it is important to note that the limited number of studies in each stratum may have led to underpowered meta-regression results. This emphasizes the necessity for future research to enhance sample sizes for more reliable findings.

**TABLE 2:** Subgroup meta-analyses for the Mediterranean diet during the perinatal period on gestational diabetes mellitus, pregnancy-induced hypertension, preterm delivery, and birth weight in cohort studies

Outcomes	Confounder			Geographical location		
	Adjusted^1^	Unadjusted	*P* value^2^	Mediterranean	Non-Mediterranean	*P* value^2^
Gestational diabetes mellitus^3^	2	5	0.37	3	4	0.90
	0.72 (0.58, 0.90)	0.91 (0.68, 1.23)		0.88 (0.52, 1.49)	0.81 (0.65, 1.02)	
Pregnancy-induced hypertension^3^	3	2	0.64	2	3	0.64
	0.73 (0.64, 0.83)	0.51 (0.16, 1.60)		0.51 (0.16, 1.60)	0.73 (0.64, 0.83)	
Preterm delivery^3^	3	2	0.45	0	5	—
	0.60 (0.40, 0.89)	0.74 (0.37, 1.49)		—	0.67 (0.49, 0.91)	
Birth weight (g)^4^	—	—	—	2	3	0.60
	—	—		27.45 (−9.48, 64.37)	50.73 (14.03, 87.44)	

Subgroup analyses were performed only for outcomes including ≥5 studies.

Abbreviations: CI, confidence interval; OR, odds ratio; WMD, weighted mean difference.

1 Includes all studies that adjusted for confounders in the analysis phase.

2 *P* value of meta-regression analysis (*P* > 0.05).

3 Values are ORs (95% CIs).

4 Values are WMD (95% CIs).

## Discussion

This meta-analysis, based on data from 23 studies involving over 100,000 pregnancies, provides a comprehensive analysis of the relationship between the maternal MD and various perinatal outcomes. Three key conclusions can be drawn from our findings. First, our overall pooled estimates reveal significant benefits of the maternal MD that has substantial beneficial effects for GDM and SGA in RCTs. Second, in cohort studies, the MD demonstrates significant positive effects on 7 of the 9 perinatal outcomes under investigation, which include GDM, pregnancy-induced hypertension, pre-eclampsia, preterm delivery, low birth weight, intrauterine growth restriction, and gestational age at delivery. Finally, meta-regression analyses did not identify the adjustment for confounders and geographical location as predictive factors for heterogeneity. This evidence can potentially guide recommendations and influence future healthcare and nutritional guidelines, promoting adherence to the MD during pregnancy.

Previous systematic reviews have explored the impact of a maternal MD during pregnancy and its associated outcomes [24,25]. One qualitative review [24] focused on studies published after 2018, with a particular focus on common pregnancy and delivery issues and outcomes assessments. It conducted a meta-analysis of 4 RCT studies that demonstrated the beneficial effects of the MD on GDM but found no significant associations for pre-eclampsia, preterm delivery, or neonatal health [25]. Another meta-analysis, based on 113 publications from 51 cohort studies concluded that Mediterranean dietary patterns were linked to a reduced risk of SGA [57]. However, there has not been a meta-analysis available that combines RCTs and cohort studies to examine the relationship between an MD during pregnancy and pregnancy outcomes. Our meta-analysis solely focuses on robust evidence from RCTs and cohort studies, thus building upon previous research and providing a comprehensive perspective on the potential associations between the maternal MD and both maternal and offspring outcomes.

Our review, summarizing data from RCTs and cohort studies, presents a growing body of evidence supporting a causal link between the MD during pregnancy and perinatal outcomes. Further stratified analyses indicate that the pooled estimates of the association between the MD during pregnancy and GDM, pregnancy-induced hypertension, and preterm delivery remain consistent within the fully adjusted subgroups, although not in the fully unadjusted subgroup. In the RCTs, 3 of 5 studies adjusted for confounding variables; however, because of the limited number of included studies, stratified analyses were not performed. Among the cohort studies, 10 of 18 (55.5%) adjusted for various confounding factors such as maternal age, pre-pregnancy BMI, education, energy intake, and smoking. However, 8 of these studies (44.4%) did not adjust for confounders. Importantly, the inclusion of studies that did not adjust for confounders did not significantly impact the overall results. This approach allowed us to combine evidence from various sources, potentially capturing a wider array of findings. For instance, results from the INMA (INfanciay Medio Ambiente, Spain) and Rhea (Greece) mother-child cohort studies [40] suggested that high adherence to the MD increased birth weight and length in smoking mothers but did not yield the same effects in non-smoking mothers. The evidence suggests that the association may differ between studies that did and did not account for potential confounding variables, which could be a significant source of heterogeneity in meta-analyses of observational studies [58]. Therefore, integrating diverse types of evidence into research syntheses may provide more comprehensive and objective conclusions that better guide for clinical practice.

The results from RCTs indicate a significant favorable association between the MD for pregnant females and GDM, as well as SGA. Moreover, significant beneficial impacts were observed for 7 of the 9 perinatal outcomes studied, including GDM, pregnancy-induced hypertension, pre-eclampsia, preterm delivery, low birth weight, intrauterine growth restriction, and gestational age at delivery. In nutritional studies, case-control and cross-sectional studies are generally more susceptible to recall bias and reverse causation compared with cohort studies. Therefore, we strongly recommend conducting more well-designed cohort studies and high-quality trials to explore related perinatal outcomes in the future.

The MD is rich in omega-3 PUFAs, vitamins, minerals, antioxidants, and polyphenols, which could explain how it mitigates adverse perinatal outcomes. One theory is that this diet’s anti-inflammatory and antioxidative properties play a crucial role. In a subset of the Nurses’ Health Study [59], the MD was linked to reduced inflammation markers (IL-6 and C-reactive protein) and endothelial dysfunction, even after adjusting for traditional CVD risk factors. This finding [60] was further confirmed by a meta-analysis of 17 trials involving 2300 patients, which identified the MD as the first dietary pattern associated with significant reductions in both proinflammatory and cytokine levels in a clinical trial [61]. In addition, this diet, characterized by a plant-based, low-carbohydrate, low-glycemic index, high-fiber, and high-protein components, promotes satiety and a sense of fullness, aiding in weight control and obesity prevention [62]. These key components may also positively impact the gut microbiota-immune system [13]. A healthy gut microbiota-immune system during pregnancy could reduce endotoxin levels and systemic inflammation, potentially lowering risk of preterm labor [63], elevated blood pressure [64], and the development of gestational diabetes [65]. Therefore, based on microbial health, adherence to the MD during pregnancy could potentially alleviate adverse pregnancy outcomes.

### Strengths and limitations

There are several strengths to this meta-analysis. First, it includes more articles than previous meta-analyses and covers a wider range of perinatal outcomes, enabling more comprehensive comparisons among these outcomes through different subgroup analyses. This makes it more robust than individual studies. Second, by providing separate estimates for RCTs and cohort studies and considering potential confounding, it yields comparable results across different study designs for most indices. Furthermore, the diversity in terms of country, race/ethnicity, study design, age, diet assessment, and MD assessment in these studies enhances the generalizability of these findings to other populations.

However, some limitations should be acknowledged. First, most studies used FFQ with varying versions to assess the MD, which may have led to inaccuracies in assessments or records. Second, the MD is not a uniform eating pattern, and regional variations and differences in score items can introduce heterogeneity, making it challenging to determine the MD score accurately. Third, most studies assessed diet at a single time point and did not consider potential changes in diet quality over time, which may be associated with the development of perinatal outcomes. Fourth, the scoring indexes for the MD have limitations, such as variability in choosing cutoff points and variations in food group distributions across different populations. Fifth, combining quantitatively adjusted and unadjusted findings, the results should be interpreted cautiously because unadjusted confounders can lead to spurious associations. Finally, some pregnancy outcomes were not clearly defined in the studies included in our analysis. For example, the definition of gestational diabetes varied across studies, and some did not specify the criteria used. Consequently, we considered gestational diabetes as an outcome based on the clinical diagnosis in the studies, which may introduce potential bias into the analysis. Given these limitations, future prospective cohort studies with uniform dietary pattern measurements, larger sample sizes from diverse populations and regions, as well as consistent score items, and even population-based intervention studies that account for a wide range of potential confounders are needed to provide a more comprehensive understanding of the association between the MD and perinatal health.

## Conclusions

Our systematic review suggests that the maternal MD may have a positive impact on perinatal outcomes. Further research is required to investigate the influence of the maternal MD on these outcomes, considering a broader range of confounding factors, to arrive at more conclusive findings.

## Author contributions

The authors’ responsibilities were as follows—JRX, HXW, PZ: designed the research; JRX, HXW, JFB, MX, NJ, WL: conducted the search, completed data collection, and performed quality assessment; JRX, HXW, JFB, WJY: analyzed data; JRX, HXW: wrote the manuscript with editorial assistance from all coauthors; JRX, HXW, PZ: critically reviewed and revised the manuscript, and interpreted the data; PZ: had primary responsibility for final content; and all authors: read and approved the final manuscript.

## Conflict of interest

The authors report no conflicts of interest.

## Funding

This research received financial support from the National Natural Science Foundation of China (82373580, 82173531), the National Key R&D Program of China (2022YFC2702901), the Research Founds of Center for Big Data and Population Health of IHM (JKS2022019), and the Foundation for Scientific Research Improvement of Anhui Medical University (2021xkjT009). The sponsors had no role in the design and conduct of the study; collection, management, analysis, and interpretation of the data; preparation, review, or approval of the manuscript; and decision to submit the manuscript for publication.

## Appendix A Supplementary data

Multimedia component1 Multimedia component1
