# Supplementary material for: Association between the Maternal Mediterranean Diet and Perinatal Outcomes: A Systematic Review and Meta-Analysis
Source: Adv Nutr. 2023 Dec 1;15(2):100159. doi: 10.1016/j.advnut.2023.100159 (PMC10801312; doi:10.1016/j.advnut.2023.100159)
Supplement: Multimedia component1 [file mmc1.docx]

**Association between the maternal Mediterranean diet and Perinatal Outcomes: A Systematic Review and Meta-Analysis**

Ji-Rong Xu et al.

**Online Supplementary Material**

**Contents**

(Supplementary data includes 5 supplementary Tables and 6 supplementary Figures.)

**Supplementary Table 1. Search strategies and results used for online databases.**

**Supplementary Table 2. Modified Newcastle-Ottawa Quality Assessment Scale for Cohort Studies.**

**Supplementary Table 3. Reason for exclusion of retrieved articles.**

**Supplementary Table 4. Characteristics of the RCTs in the meta-analysis of maternal Mediterranean diet and perinatal outcomes.**

**Supplementary Table 5. Characteristics of the cohort studies in the meta-analysis of maternal Mediterranean diet and perinatal outcomes.**

**Supplementary Figure 1. Cochrane risk of bias (graph) for RCTs.**

**Supplementary Figure 2. Meta-analysis of the maternal Mediterranean diet and adverse perinatal outcomes (RCTs).**

**Supplementary Figure 3. Meta-analysis of the maternal Mediterranean diet and adverse perinatal outcomes (cohort studies).**

**Supplementary Figure 4. Subgroup analysis based on the adjustment for confounders for GDM, pregnancy-induced hypertension, and preterm delivery (cohort studies).**

**Supplementary Figure 5. Subgroup analysis based on the geographical location of GDM, pregnancy-induced hypertension, preterm delivery, and birth weight (g) (cohort studies).**

**Supplementary Figure 6. Sensitivity analysis by omitting individual studies (cohort studies).**

**Supplementary Table 1. Search strategies and results used for online databases.**

| **Database**  **10/3/2023** | **Search Terms** | **Results**  **Yielded** |
| --- | --- | --- |
| PubMed | ((Diet, Mediterranean[Title/Abstract]) OR (Mediterranean Diet[Title/Abstract]) OR (Diets, Mediterranean[Title/Abstract]) OR (Mediterranean Diets[Title/Abstract]) OR (Dietary pattern[Title/Abstract]) OR (Dietary patterns[Title/Abstract])) AND ((neonatal*[Title/Abstract]) OR (neonate*[Title/Abstract]) OR (birth*[Title/Abstract]) OR (pregnan*[Title/Abstract]) OR (prenatal[Title/Abstract]) OR (prenatal*[Title/Abstract]) OR (perinatal[Title/Abstract]) OR (perinatal*[Title/Abstract]) OR (antenatal[Title/Abstract]) OR (antenatal*[Title/Abstract]) OR (maternal[Title/Abstract]) OR (gestation*[Title/Abstract]) OR (antepartum[Title/Abstract]) OR (antepartum*[Title/Abstract]) OR (deliver*[Title/Abstract]) OR (periconception*[Title/Abstract]) OR (periconception[Title/Abstract])) AND (Human[Filters]) | 1266 |
| Web of Science | ((((((TS=(Diet, Mediterranean)) OR TS=(Mediterranean Diet)) OR TS=(Diets, Mediterranean)) OR TS=(Mediterranean Diets)) OR TS=(Dietary pattern)) OR TS=(Dietary patterns)) AND ((((((((((((((((TS=(neonatal*)) OR TS=(neonate*)) OR TS=(birth*)) OR TS=(newbirth*)) OR TS=(pregnan*)) OR TS=(prenatal)) OR TS=(prenatal*)) OR TS=(perinatal)) OR TS=(perinatal*)) OR TS=(antenatal)) OR TS=(antenatal*)) OR TS=(maternal)) OR TS=(gestation*)) OR TS=(antepartum)) OR TS=(antepartum*)) OR TS=(deliver*)) OR TS=(periconception*)) OR TS=(periconception)) AND Pregnancy (MeSH Terms) | 3174 |
| Cochrane Library | 'Diet, Mediterranean':ti,ab,kw OR 'Mediterranean Diet':ti,ab,kw OR 'Diets, Mediterranean':ti,ab,kw OR 'Mediterranean Diets':ti,ab,kw OR 'Dietary pattern':ti,ab,kw OR 'Dietary patterns':ti,ab,kw AND 'neonatal*':ti,ab,kw OR 'neonate*':ti,ab,kw OR 'birth*':ti,ab,kw OR 'newbirth*':ti,ab,kw OR 'pregnan*':ti,ab,kw OR 'prenatal':ti,ab,kw OR 'prenatal*':ti,ab,kw OR 'perinatal':ti,ab,kw OR 'perinatal*':ti,ab,kw OR 'antenatal':ti,ab,kw OR 'antenatal*':ti,ab,kw OR 'maternal':ti,ab,kw OR 'gestation*':ti,ab,kw OR 'antepartum':ti,ab,kw OR 'antepartum*':ti,ab,kw OR 'deliver*':ti,ab,kw OR 'periconception*':ti,ab,kw OR 'periconception':ti,ab,kw | 685 |
| Manual search | NA | 3 |

Abbreviations: n/a, no application.

**Supplemental Table 2. Modified Newcastle-Ottawa Quality Assessment Scale for the Observational Studies.**

| **Author*** | **Study** | **Selection^1^** | | | | **Comparability^2^** | | **Outcome^3^** | | | **Score** |
| --- | --- | --- | --- | --- | --- | --- | --- | --- | --- | --- | --- |
| Chatzi et al., 2012 (6) | INMA (Spain) and RHEA (Greece) mother-child cohort studies | 1 | 1 | 1 | 1 | 1 | 1 | 1 | 1 | 1 | 9 |
| Gonzalez-Nahm et al., 2020 (7) | Mother–child cohort | 1 | 1 | 0 | 0 | 1 | 1 | 1 | 1 | 0 | 6 |
| Haugen et al., 2008 (8) | Mother–child cohort, (the Norwegian Mother-Child Cohort Study) (MoBa) | 1 | 1 | 0 | 1 | 1 | 1 | 1 | 1 | 1 | 8 |
| Mikkelsen et al., 2008 (9) | Birth cohort (the Danish National Birth Cohort) | 1 | 1 | 0 | 1 | 1 | 1 | 1 | 1 | 1 | 8 |
| Minhas et al., 2022 (10) | Prospective cohort study (the Boston Birth Cohort) | 1 | 1 | 1 | 1 | 1 | 1 | 1 | 1 | 1 | 9 |
| Monthe-Dreze et al., 2021 (11) | Prospective cohort study | 1 | 1 | 1 | 1 | 0 | 0 | 1 | 1 | 0 | 6 |
| Papazian et al., 2019 (12) | Cross-sectional study | 1 | 1 | 1 | 0 | 0 | 0 | 1 | 0 | 1 | 5 |
| Schoenaker et al., 2016 (13) | Cohort study (The Australian Longitudinal Study on Women’s Health) | 0 | 1 | 0 | 1 | 1 | 1 | 1 | 1 | 1 | 7 |
| Timmermans et al., 2011 (14) | Prospective cohort study (the Generation R Study) | 1 | 1 | 0 | 1 | 1 | 1 | 1 | 1 | 0 | 7 |
| Timmermans et al., 2012 (15) | Prospective cohort study (the Generation R Study) | 1 | 1 | 0 | 1 | 1 | 1 | 1 | 1 | 0 | 7 |
| Yisahak et al., 2021 (16) | Prospective cohort study (National Institute of Child Health and Human Development) (NICHD). | 1 | 1 | 0 | 1 | 1 | 1 | 1 | 1 | 1 | 8 |
| Li et al., 2021 (17) | Prospective cohort study (National Institute of Child Health and Human Development (NICHD) ). | 1 | 1 | 0 | 1 | 1 | 1 | 1 | 1 | 1 | 8 |
| Diaz-Lopez et al., 2022 (18) | Mother–child cohort study (ECLIPSES) | 1 | 1 | 1 | 1 | 1 | 1 | 1 | 1 | 1 | 9 |
| Ashwin et al.,2022 (19) | Birth cohort (ORIGINS) | 1 | 1 | 0 | 1 | 1 | 1 | 1 | 1 | 1 | 8 |
| Fernandez-Barres et al., 2019 (20) | Birth cohort(INMA)（Infancia Medio Ambiente） | 1 | 1 | 0 | 1 | 1 | 1 | 1 | 1 | 1 | 8 |
| Mantzoros et al., 2010 (21) | Prospective cohort study | 1 | 1 | 0 | 1 | 1 | 1 | 1 | 1 | 1 | 8 |
| Karamanos et al., 2014 (22) | Prospective observational study | 0 | 1 | 1 | 1 | 1 | 1 | 1 | 1 | 1 | 8 |
| Bedard et al.,2020 (23) | Birth cohort (ALSPAC) (the Avon Longitudinal Study of Parents and Children) | 1 | 1 | 1 | 1 | 1 | 1 | 1 | 1 | 1 | 9 |

^*^Author： References are based on the supplement file.

^1^ Selection: 1) Representativeness of the exposed cohort (truly/somewhat representative of the exposure in the community, 1; selected group or no description, 0);

2) Selection of the non-exposed cohort (drawn from the same community as the exposed cohort, 1; drawn from a different source or no description, 0);

3) Ascertainment of exposure (secure record or structured interview, 1; self-report or no description, 0);

4) Demonstration that outcome of interest was not present at start (yes,1; no, 0);

*In the case of CVD mortality studies, outcome of interest is still the presence of CVD incidence);

^2^ Comparability: 1a) study controls for dietary pattern (yes,1; no, 0);

1b) study controls for any additional factor (yes,1; no, 0);

^3^ Outcome: 1) Assessment of outcome (independent blind assessment, 1; self-report or no description, 0);

2) Was follow-up long enough (≥5 years) for outcomes to occur (yes,1; no, 0);

3) Adequacy of follow up of cohorts (≥80%) (yes,1; no or no statement, 0).

**Supplementary Table 3. Reason for exclusion of retrieved articles.**

| References | Reason for exclusion |
| --- | --- |
| 1. de la Torre NG, Assaf-Balut C, Jiménez Varas I, Del Valle L, Durán A, Fuentes M, et al. Effectiveness of Following Mediterranean Diet Recommendations in the Real World in the Incidence of Gestational Diabetes Mellitus (GDM) and Adverse Maternal-Foetal Outcomes: A Prospective, Universal, Interventional Study with a Single Group. The St Carlos Study. Nutrients. 2019 May 28;11(6):1210. 2. Chong MF, Godfrey KM, Gluckman P, Tan KH, Shek LP, Meaney M, et al. Influences of the perinatal diet on maternal and child health: insights from the GUSTO study. Proc Nutr Soc. 2020 Aug;79(3):253-258. 3. Trak-Fellermeier MA, Campos M, Meléndez M, Pomeroy J, Palacios C, Rivera-Viñas J, et al. PEARLS randomized lifestyle trial in pregnant Hispanic women with overweight/obesity: gestational weight gain and offspring birthweight. Diabetes Metab Syndr Obes. 2019 Feb 18;12:225-238. 4. Incidence of gestational diabetes mellitus (GDM) and the Mediterranean diet. Cochrane Library. https://trialsearch.who.int/Trial2.aspx?TrialID=ISRCTN16896947, 2016. 5. Mediterranean diet to prevent type 2 diabetes in mothers who had diabetes in pregnancy: a feasibility study. Cochrane Library. https://trialsearch.who.int/Trial2.aspx?TrialID=ISRCTN40582975, 2019. 6. REduction in the incidence of GEstational DIAbetes mellitus (GDM) with MEDDiet/Lifestyle. Cochrane Library. https://trialsearch.who.int/Trial2.aspx?TrialID=ISRCTN84389045, **2013.** 7. M Landry, L Aronica, C Gardner, S Kim, J Robinson, A Chang, et al. Effect of a ketogenic diet vs mediterranean diet on hba1c: the keto-med randomized crossover trial. Obesity (Silver Spring, Md.), 2021, 29(SUPPL 2), 86. 8. Effectiveness of Mediterranean Diet Intervention in Pregnant Women. Cochrane Database of Systematic Reviews.2023 May;12(3). 9. Tobias DK, Zhang C, Chavarro J, Olsen S, Bao W, Bjerregaard AA, et al. Healthful dietary patterns and long-term weight change among women with a history of gestational diabetes mellitus. Int J Obes (Lond). 2016 Nov;40(11):1748-1753. 10. Al Wattar BH, Dodds J, Placzek A, Spyreli E, Moore A, Hooper R, et al. Effect of simple, targeted diet in pregnant women with metabolic risk factors on maternal and fetal outcomes (ESTEEM): study protocol for a pragmatic multicentre randomised trial. BMJ Open. 2016 Oct 21;6(10):e013495. 11. Crovetto F, Crispi F, Borras R, Paules C, Casas R, Martín-Asuero A, et al. Mediterranean diet, Mindfulness-Based Stress Reduction and usual care during pregnancy for reducing fetal growth restriction and adverse perinatal outcomes: IMPACT BCN (Improving Mothers for a better Prenatal Care Trial BarCeloNa): a study protocol for a randomized controlled trial. Trials. 2021 May 24;22(1):362. 12. Liu ST, Lin CC, Wei JC. Mediterranean Diet or Mindfulness-Based Stress Reduction and Prevention of Small-for-Gestational-Age Birth Weights in Newborns. JAMA. 2022 Apr 5;327(13):1292-1293. 13. Crovetto F, Crispi F, Gratacós E. Mediterranean Diet or Mindfulness-Based Stress Reduction and Prevention of Small-for-Gestational-Age Birth Weights in Newborns-Reply. JAMA. 2022 Apr 5;327(13):1293-1294. 14. Amati F, Hassounah S, Swaka A. The Impact of Mediterranean Dietary Patterns During Pregnancy on Maternal and Offspring Health. Nutrients. 2019 May 17;11(5):1098. 15. Barger MK. Maternal nutrition and perinatal outcomes. J Midwifery Womens Health. 2010 Nov-Dec;55(6):502-11. 16. Chen X, Zhao D, Mao X, Xia Y, Baker PN, Zhang H. Maternal Dietary Patterns and Pregnancy Outcome. Nutrients. 2016 Jun 7;8(6):351. 17. Fedullo AL, Schiattarella A, Morlando M, Raguzzini A, Toti E, De Franciscis P, et al. Mediterranean Diet for the Prevention of Gestational Diabetes in the Covid-19 Era: Implications of Il-6 In Diabesity. Int J Mol Sci. 2021 Jan 26;22(3):1213. 18. Fischer M, Stronati M, Lanari M. Mediterranean diet, folic acid, and neural tube defects. Ital J Pediatr. 2017 Aug 17;43(1):74. 19. Hassani Zadeh S, Boffetta P, Hosseinzadeh M. Dietary patterns and risk of gestational diabetes mellitus: A systematic review and meta-analysis of cohort studies. Clin Nutr ESPEN. 2020 Apr;36:1-9. 20. Mijatovic-Vukas J, Capling L, Cheng S, Stamatakis E, Louie J, Cheung NW, et al. Associations of Diet and Physical Activity with Risk for Gestational Diabetes Mellitus: A Systematic Review and Meta-Analysis. Nutrients. 2018 May 30;10(6):698. 21. Netting MJ, Middleton PF, Makrides M. Does maternal diet during pregnancy and lactation affect outcomes in offspring? A systematic review of food-based approaches. Nutrition. 2014 Nov-Dec;30(11-12):1225-41. 22. Zhang Y, Xia M, Weng S, Wang C, Yuan P, Tang S. Effect of Mediterranean diet for pregnant women: a meta-analysis of randomized controlled trials. J Matern Fetal Neonatal Med. 2022 Dec;35(24):4824-4829. | No original data |
| 1. Bellati U, Pompa P, Liberati M. Valutazione dell'influenza di una dieta "mediterranea" e della massa corporea pregravidica sull'accrescimento intrauterino [Evaluation of the effect of a "Mediterranean diet" and pre-pregnancy body mass on fetal growth]. Minerva Ginecol. 1995 Jun;47(6):259-62. Italian. 2. Corsi Decenti E, Zambri F, Salvatore MA, Lacaria E, Chellini M, Grillo A, et al. Dietary habits, lifestyle, and gestational diabetes in immigrant women: a survey in Northwestern Tuscany (Central Italy). Epidemiol Prev. 2022 Jul-Aug;46(4):259-267. English. 3. Schlienger J‐L, Monnier L. Mediterranean diets and diabetes prevention: time for evidence. Medecine des maladies metaboliques, 2020, 14(7), 626‐631 | No full-text |
| 1. Assaf-Balut C, García de la Torre N, Duran A, Fuentes M, Bordiú E, Del Valle L, et al. A Mediterranean Diet with an Enhanced Consumption of Extra Virgin Olive Oil and Pistachios Improves Pregnancy Outcomes in Women Without Gestational Diabetes Mellitus: A Sub-Analysis of the St. Carlos Gestational Diabetes Mellitus Prevention Study. Ann Nutr Metab. 2019;74(1):69-79. 2. Assaf-Balut C, García de la Torre N, Durán A, Fuentes M, Bordiú E, Del Valle L, et al. A Mediterranean diet with additional extra virgin olive oil and pistachios reduces the incidence of gestational diabetes mellitus (GDM): A randomized controlled trial: The St. Carlos GDM prevention study. PLoS One. 2017 Oct 19;12(10):e0185873. 3. Assaf-Balut C, García de la Torre N, Fuentes M, Durán A, Bordiú E, Del Valle L, et al. A High Adherence to Six Food Targets of the Mediterranean Diet in the Late First Trimester is Associated with a Reduction in the Risk of Materno-Foetal Outcomes: The St. Carlos Gestational Diabetes Mellitus Prevention Study. Nutrients. 2018 Dec 31;11(1):66. 4. de la Torre NG, Assaf-Balut C, Jiménez Varas I, Del Valle L, Durán A, Fuentes M, et al. Effectiveness of Following Mediterranean Diet Recommendations in the Real World in the Incidence of Gestational Diabetes Mellitus (GDM) and Adverse Maternal-Foetal Outcomes: A Prospective, Universal, Interventional Study with a Single Group. The St Carlos Study. Nutrients. 2019 May 28;11(6):1210. 5. Gesteiro E, Rodríguez Bernal B, Bastida S, Sánchez-Muniz FJ. Maternal diets with low healthy eating index or Mediterranean diet adherence scores are associated with high cord-blood insulin levels and insulin resistance markers at birth. Eur J Clin Nutr. 2012 Sep;66(9):1008-15. 6. Melero V, Assaf-Balut C, Torre NG, Jiménez I, Bordiú E, Valle LD, et al. Benefits of Adhering to a Mediterranean Diet Supplemented with Extra Virgin Olive Oil and Pistachios in Pregnancy on the Health of Offspring at 2 Years of Age. Results of the San Carlos Gestational Diabetes Mellitus Prevention Study. J Clin Med. 2020 May 13;9(5):1454. 7. Peraita-Costa I, Llopis-González A, Perales-Marín A, Sanz F, Llopis-Morales A, Morales-Suárez-Varela M. A Retrospective Cross-Sectional Population-Based Study on Prenatal Levels of Adherence to the Mediterranean Diet: Maternal Profile and Effects on the Newborn. Int J Environ Res Public Health. 2018 Jul 19;15(7):1530. 8. Rhee DK, Ji Y, Hong X, Pearson C, Wang X, Caulfield LE. Mediterranean-Style Diet and Birth Outcomes in an Urban, Multiethnic, and Low-Income US Population. Nutrients. 2021 Apr 3;13(4):1188. 9. Schoenaker DA, Soedamah-Muthu SS, Callaway LK, Mishra GD. Prepregnancy dietary patterns and risk of developing hypertensive disorders of pregnancy: results from the Australian Longitudinal Study on Women's Health. Am J Clin Nutr. 2015 Jul;102(1):94-101. 10. Schoenaker DA, Soedamah-Muthu SS, Callaway LK, Mishra GD. Pre-pregnancy dietary patterns and risk of gestational diabetes mellitus: results from an Australian population-based prospective cohort study. Diabetologia. 2015 Dec;58(12):2726-35. 11. H Al Wattar B, Dodds J, Placzek A, Beresford L, Spyreli E, Moore A, et al. Mediterranean-style diet in pregnant women with metabolic risk factors (ESTEEM): A pragmatic multicentre randomised trial. PLoS Med. 2019 Jul 23;16(7):e1002857. 12. Gonzalez-Nahm S, Mendez M, Robinson W, Murphy SK, Hoyo C, Hogan V, et al. Low maternal adherence to a Mediterranean diet is associated with increase in methylation at the *MEG3-IG* differentially methylated region in female infants. Environ Epigenet. 2017 May 6;3(2):dvx007. | Article with similar exposure and outcome variable (duplicates) |
| 1. Silva-del Valle MA, Sánchez-Villegas A, Serra-Majem L. Association between the adherence to the Mediterranean diet and overweight and obesity in pregnant women in Gran Canaria. Nutr Hosp. 2013 May-Jun;28(3):654-9. 2. Chatzi L, Rifas-Shiman SL, Georgiou V, Joung KE, Koinaki S, Chalkiadaki G, et al. Adherence to the Mediterranean diet during pregnancy and offspring adiposity and cardiometabolic traits in childhood. Pediatr Obes. 2017 Aug;12 Suppl 1(Suppl 1):47-56. 3. Di Renzo L, Marchetti M, Rizzo G, Gualtieri P, Monsignore D, Dominici F, et al. Adherence to Mediterranean Diet and Its Association with Maternal and Newborn Outcomes. Int J Environ Res Public Health. 2022 Jul 12;19(14):8497. 4. Koutelidakis AE, Alexatou O, Kousaiti S, Gkretsi E, Vasios G, Sampani A, et al. Higher adherence to Mediterranean diet prior to pregnancy is associated with decreased risk for deviation from the maternal recommended gestational weight gain. Int J Food Sci Nutr. 2018 Feb;69(1):84-92. 5. Mahjoub F, Ben Jemaa H, Ben Sabeh F, Ben Amor N, Gamoudi A, Jamoussi H. Impact of nutrients and Mediterranean diet on the occurrence of gestational diabetes. Libyan J Med. 2021 Dec;16(1):1930346. 6. Šarac J, Havaš Auguštin D, Lovrić M, Stryeck S, Šunić I, Novokmet N, et al. A Generation Shift in Mediterranean Diet Adherence and Its Association with Biological Markers and Health in Dalmatia, Croatia. Nutrients. 2021 Dec 20;13(12):4564. 7. Landry MJ, Crimarco A, Perelman D, Durand LR, Petlura C, Aronica L, et al. Adherence to Ketogenic and Mediterranean Study Diets in a Crossover Trial: The Keto-Med Randomized Trial. Nutrients. 2021 Mar 17;13(3):967. 8. Lindsay KL, Buss C, Wadhwa PD, Entringer S. The Effect of a Maternal Mediterranean Diet in Pregnancy on Insulin Resistance is Moderated by Maternal Negative Affect. Nutrients. 2020 Feb 6;12(2):420. 9. Babili MG, Amerikanou C, Papada E, Christopoulos G, Tzavara C, Kaliora AC. The effect of prenatal maternal physical activity and lifestyle in perinatal outcome: results from a Greek study. Eur J Public Health. 2020 Apr 1;30(2):328-332. 10. Cano-Ibáñez N, Martínez-Galiano JM, Luque-Fernández MA, Martín-Peláez S, Bueno-Cavanillas A, Delgado-Rodríguez M. Maternal Dietary Patterns during Pregnancy and Their Association with Gestational Weight Gain and Nutrient Adequacy. Int J Environ Res Public Health. 2020 Oct 28;17(21):7908. 11. Mariscal-Arcas M, Lopez-Martinez C, Granada A, Olea N, Lorenzo-Tovar ML, Olea-Serrano F. Organochlorine pesticides in umbilical cord blood serum of women from Southern Spain and adherence to the Mediterranean diet. Food Chem Toxicol. 2010 May;48(5):1311-5. 12. Ramos-Levi A, Barabash A, Valerio J, García de la Torre N, Mendizabal L, Zulueta M, et al. Genetic variants for prediction of gestational diabetes mellitus and modulation of susceptibility by a nutritional intervention based on a Mediterranean diet. Front Endocrinol (Lausanne). 2022 Oct 13;13:1036088. 13. Spadafranca A, Piuri G, Bulfoni C, Liguori I, Battezzati A, Bertoli S, et al. Adherence to the Mediterranean Diet and Serum Adiponectin Levels in Pregnancy: Results from a Cohort Study in Normal Weight Caucasian Women. Nutrients. 2018 Jul 20;10(7):928. 14. Chatzi L, Rifas-Shiman SL, Georgiou V, Joung KE, Koinaki S, Chalkiadaki G,et al. Adherence to the Mediterranean diet during pregnancy and offspring adiposity and cardiometabolic traits in childhood. Pediatr Obes. 2017 Aug;12 Suppl 1(Suppl 1):47-56. 15. Bellati U, Pompa P, Liberati M. Valutazione analitica di una dieta "mediterranea" in gravidanza [Analytic evaluation of a "Mediterranean" diet in pregnancy]. Minerva Ginecol. 1994 Apr;46(4):183-7. Italian. 16. Al Wattar BH, Dodds J, Placzek A, Spyreli E, Higgins S, Moore A, et al. Mediterranean diet based intervention in pregnancy to improve maternal and fetal outcomes: Methodological challenges and lessons learned from the multicentre ESTEEM study. Contemp Clin Trials Commun. 2017 Mar 29;6:72-77. 17. Havaš Auguštin D, Šarac J, Lovrić M, Živković J, Malev O, Fuchs N, et al Adherence to Mediterranean Diet and Maternal Lifestyle during Pregnancy: Island-Mainland Differentiation in the CRIBS Birth Cohort. Nutrients. 2020 Jul 22;12(8):2179. 18. Tobias DK, Zhang C, Chavarro J, Bowers K, Rich-Edwards J, Rosner B, et al. Prepregnancy adherence to dietary patterns and lower risk of gestational diabetes mellitus. Am J Clin Nutr. 2012 Aug;96(2):289-95. 19. Saunders L, Guldner L, Costet N, Kadhel P, Rouget F, Monfort C, et al. Effect of a Mediterranean diet during pregnancy on fetal growth and preterm delivery: results from a French Caribbean Mother-Child Cohort Study (TIMOUN). Paediatr Perinat Epidemiol. 2014 May;28(3):235-44. | No sufficient data |
| 1. Izadi V, Tehrani H, Haghighatdoost F, Dehghan A, Surkan PJ, Azadbakht L. Adherence to the DASH and Mediterranean diets is associated with decreased risk for gestational diabetes mellitus. Nutrition. 2016 Oct;32(10):1092-6. 2. Olmedo-Requena R, Gómez-Fernández J, Amezcua-Prieto C, Mozas-Moreno J, Khan KS, Jiménez-Moleón JJ. Pre-Pregnancy Adherence to the Mediterranean Diet and Gestational Diabetes Mellitus: A Case-Control Study. Nutrients. 2019 May 1;11(5):1003. 3. Carmichael SL, Yang W, Shaw GM; National Birth Defects Prevention Study. Maternal dietary nutrient intake and risk of preterm delivery. Am J Perinatol. 2013 Aug;30(7):579-88. 4. Martínez-Galiano JM, Olmedo-Requena R, Barrios-Rodríguez R, Amezcua-Prieto C, Bueno-Cavanillas A, Salcedo-Bellido I, et al. Effect of Adherence to a Mediterranean Diet and Olive Oil Intake during Pregnancy on Risk of Small for Gestational Age Infants. Nutrients. 2018 Sep 5;10(9):1234. 5. Peraita-Costa I, Llopis-González A, Perales-Marín A, Diago V, Soriano JM, Llopis-Morales A, et al. Maternal profile according to Mediterranean diet adherence and small for gestational age and preterm newborn outcomes. Public Health Nutr. 2021 Apr;24(6):1372-1384. | Case-control studies |
| 1. Gesteiro E, Bastida S, Rodríguez Bernal B, Sánchez-Muniz FJ. Adherence to Mediterranean diet during pregnancy and serum lipid, lipoprotein and homocysteine concentrations at birth. Eur J Nutr. 2015 Oct;54(7):1191-9. 2. Papazian T, Serhal A, Hout H, Younes H, Tayeh GA, Azouri J, et al. Discrepancies among different tools evaluating Mediterranean diet adherence during pregnancy, correlated to maternal anthropometric, dietary and biochemical characteristics. Clin Nutr. 2019 Jun;38(3):1398-1405. 3. Tomaino L, Reyes Suárez D, Reyes Domínguez A, García Cruz LM, Ramos Díaz M, Serra Majem L. Adherence to Mediterranean diet is not associated with birthweight - Results form a sample of Canarian pregnant women. Nutr Hosp. 2020 Feb 17;37(1):86-92. English. 4. De Giuseppe R, Bocchi M, Maffoni S, Del Bo E, Manzoni F, Cerbo RM, et al. Mediterranean Diet and Lifestyle Habits during Pregnancy: Is There an Association with Small for Gestational Age Infants? An Italian Single Centre Experience. Nutrients. 2021 Jun 5;13(6):1941. | Cross-sectional studies |

**Supplementary Table 4. Characteristics of RCTs in the meta-analysis of maternal Mediterranean diet and perinatal outcomes.**

| **Study** | | | **Mediterranean group** | | | **Control** | | | **Exposure** | | **Outcomes** | |
| --- | --- | --- | --- | --- | --- | --- | --- | --- | --- | --- | --- | --- |
| **Reference^1^** | **Country** | **Race/ethnicity** | **No** | **Age (years)** | **Methods** | **No** | **Age (years)** | **Methods** | **Diet assessment** | **MD assessment** | **Measured outcomes and results** | **Covariate^2^** |
| Al Wattar, 2019 (1) | United Kingdom | White, Asian, Black, Other | 593 | 31.4±5.2 | Mediterranean-style diet with high intake of nuts, extra virgin olive oil, fruits, vegetables, non-refined grains, and legumes; moderate to high consumption of fish; low to moderate intake of poultry and daily products; low intake of red and processed meat; and avoidance of sugary drinks, fat food, and food rich in animal fat | 612 | 30.9±5.2 | Standard diet | 24-Hour Day Record and FFQ | ESTEEM Q (a validated FFQ for MED and a short questionary) | GDM, preeclampsia, SGA, LGA, PTD, NICU admission, CS, birth weight (g) | 1,2,3,4,5 |
| Crovetto et al., 2021 (2) | Spain | Asian, Black, Latin American, Maghreb, White. | 392 | 37.2±4.4 | Mediterranean-style diet with high intake of nuts, extra virgin olive oil, fruits, vegetables, non-refined grains, and legumes; moderate to high consumption of fish; low to moderate intake of poultry and daily products; low intake of red and processed meat; and avoidance of sugary drinks, fat food, and food rich in animal fat | 401 | 37±5.4 | Standard diet | 151-item FFQ and 7-day dietary journal | 17-point MED score | Pre-eclampsia, PTD, SGA | NA |
| Melero et al., 2020 (3) | Spain | Hispanic, Caucasian. | 128 | 31.7±5.4 | Mediterranean diet with the consumption of EVOO (>=40 mL/day) and a handful of pistachios (25-30 g) at least 3days a week. | 132 | 31.3±5.6 | Standard diet | FFQ | 12-point MEDAS score | GDM, HDP, pre-eclampsia, CS, PTD, SGA, LGA, NICU admission, GA at delivery (weeks), Birth weight (g), Birth length (cm) | 1,6,7 |
| Zhao et al., 2022 (4) | China | NA | 250 | 28±5.2 | MED with extra EVOO and pistachios | 250 | 29.4±5.6 | MED without extra EVOO and pistachios | MED intervention | NA | GDM, HDP, pre-eclampsia, CS, PTD, SGA, LGA, NICU admission, GA at delivery (weeks), birth weight(g), birth length (cm) | 1,2,3,4,5,7,8 |
| Assaf-Balut et al.2019 (5) | Spain | Hispanic, Caucasian, others. | 999 | 33.32± 4.78 | Dietary guidelines based on a Mediterranean diet, with emphasis on the consumption of a daily intake of ≥40 mL of extra virgin olive oil and a handful of nuts,. | 676 | 33.54 ± 5.11 | standard-care dietary guidelines based on MED but limiting the consumption of fats (including extra virgin olive oil and nuts). | FFQ | 14-item MEDAS | GDM | NA |

Abbreviation: MD, Mediterranean diet; MEDAS, Mediterranean diet adherence score.

^1^References are based on the supplement file.

^2^Adjustments: age (1), history of previous gestational diabetes (2), family history of hypertensive disorders (3), family history of diabetes (4), history of stillbirth (5), parity (6), BMI (7), smoking status (8).

**Supplementary Table 5. Characteristics of the observational studies in meta-analysis of maternal Mediterranean diet and perinatal outcomes.**

| **Study** | | **Participants** | | | **Exposure** | | **Outcomes** | | |
| --- | --- | --- | --- | --- | --- | --- | --- | --- | --- |
| **Reference^1^** | **Country** | **Sample (hMED)** | **Race/ethnicity** | **Age, y, Mean ± SD** | **Diet assessment** | **MD assessment** | **Highest vs lowest categories of MD score** | **Measured outcomes and results** | **Covariate^2^** |
| Chatzi et al., 2012 (6) | Spain and Greece. | 3284 (14.3) | NA | 30.3 | 100-, 250-item FFQ | 8-point MD score | (Score 6 - 8) vs ≤(score 0 - 3) | HDP, and IUGR | 1,2,9 |
| Gonzalez-Nahm et al., 2020 (7) | American | 298 (30.9) | White, Black, Hispanic and Others. | NA | FFQ | 9-point MD score | (Score ≥4) vs (score <4) | PTD, and LBW | NA |
| Haugen et al., 2008 (8) | Norway | 26125 (2.2) | NA | 30.0±4 | FFQ | 5-criteria MD index | (All 5 criteria) vs (0 criteria) | PTD and birth weight (g) | 3,17,18,19,20 |
| Mikkelsen et al., 2008 (9) | Denmark | 35530 (3.2) | NA | 29.3±3.8 | 360-item FFQ | 4-criteria MD | (All 5 criteria) vs (0 criteria) | PTD | 3,17,18,19,20 |
| Minhas et al., 2022 (10) | American | 8507 (35.5) | White, Black, Hispanic, and Other | 28(23-33) * | FFQ | 38-point MSDS | Tertile 3 vs Tertile 1 | GDM, pre-eclampsia, and LBW | 1,9,12,17,21,22,23 |
| Monthe-Dreze et al., 2021 (11) | American | 1459 (33.8) | White, Black, Hispanic, Asia and Other. | 32.6±4.7 | 166-item FFQ | 9-point MD score | Quartile 4 vs Quartile1 | birth weight（g), and GA at delivery（weeks) | NA |
| Parlapani et al., 2019 (12) | Greece | 82 (54.9) | NA | NA | FFQ | MDA score | (≥50^th^ percentile) vs (<50^th^ percentile) | GDM, HDP, and IUGR | 1,3,6,10,12,14,17,24 |
| Schoenaker et al., 2016 (13) | Australia | 3378 (7.1) | Australian, Other English-speaking background or Europe, Asia or other | 28±1.4 | 101-item FFQ | 10-point MD score | (Score 4 - 10) vs (score 0 – 3) | GDM and HDP | 2,7,12,17,25 |
| Timmermans et al., 2011 (14) | Netherlands | 3187 (33.4) | Dutch women | 31.4±4.3 | 293-item FFQ | MDA score | Tertile 3 vs Tertile 1 | HDP and preeclampsia | 1,2,9,12,15,17,26 |
| Timmermans et al., 2012 (15) | Netherlands | 3207 (33.3) | Dutch women | 31.3±4.3 | 293-item FFQ | MDA score | Tertile 3 vs Tertile 1 | IUGR, and birth weight(g) | NA |
| Yisahak et al., 2021 (16) | American | 1948 (17.2) | Non-Hispanic white, non-Hispanic black, Hispanic, Asian/Pacific Islander. | 28.1±5.8 | FFQ | 8-point aMED score | Quartile 4 vs Quartile 1 | PTD, LBW, SGA, and LGA | 1,2,3,7,8,11,12,13,17,18,21,22,29, |
| Li et al., 2021 (17) | American | 1887 (18) | Non-Hispanic white, non-Hispanic black, Hispanic, Asian/Pacific Islander. | 28.1±5.8 | FFQ | 9-point aMED score | Quartile 4 vs Quartile 1 | GDM, HDP, pre-eclampsia, and PTD | 1,2,3,4,5,6,7,12,21,30 |
| Diaz-Lopez et al., 2022 (18) | Spain | 614 (22.1) | NA | 30.5±5.1 | FFQ | 18-point rMedDiet score | Tertile 3 vs Tertile 1 | SGA, CS, GWG (kg), birth weight (g), birth length (cm), birth HC (cm), and GA at delivery (weeks) | 1,3,9,12,19,31 |
| Ashwin et al.,2022 (19) | Australia | 458 (10.3) | Australia, New Zealand，British/Irish，European，Asian，American，and other. | 32.5±4.6 | 14-item FFQ | 13-point MDA score and PREDIMED | (Score 8–13) vs (score 0–4) | GDM, CS, birth weight (g), birth length (cm), and birth HC (cm) | NA |
| Fernandez-Barres et al., 2019 (20) | Spain | 2195 (29.1) | NA | 30.8±4.2 | 101-item FFQ | 16-point rMED score | Tertile 3 vs Tertile 1 | GDM, and Birth weight (g) | NA |
| Mantzoros et al., 2010 (21) | American | 780 (31.4) | white and nonwhite | 32.2±5.0 | FFQ | 9-point MD score | (Score 6 - 9 ) vs (score 0–3) | GWG (kg), and GA at delivery (weeks) | NA |
| Karamanos et al., 2014 (22) | 10 Mediterranean countries | 1076 (NA) | NA | 30.9±0.9 | FFQ | MedDiet Index | Tertile 3 vs Tertile 1 | GDM | NA |
| Bedard et al.,2020 (23) | UK | 8907 (61.0) | white and nonwhite | 28.9±4.6 | 43-item FFQ | 7-point MD score | (Score 4 - 7 ) vs (score 0–3) | LBW, macrosomia, and GA at delivery (weeks) | NA |

Abbreviation: DM, diabetes mellitus; GDM, gestational diabetes mellitus; HDP, pregnancy-induced hypertension; CS, cesarean section; LBW, low birth weight; SGA, small-for-gestational age fetus; LGA, large-for-gestational age fetus; PTD, preterm delivery; IUGR, intrauterine growth restriction; NICU, a neonatal intensive care unit; GWG, gestational weight gain; HC, head circumference; MEDAS, Mediterranean diet adherence screener; MDS, Mediterranean diet score; MSDS, Mediterranean‐style diet score; SFFQ, semi-quantitative FFQ; MD, Mediterranean Diet, MDS, Mediterranean Diet Score, MDA, Mediterranean Diet Adherence, FFQ, Food Frequency Questionnaire.

^1^References are based on the supplement file.

^2^Adjustments: age (1), energy intake (2), BMI (3), family history DM (3), previous DM (4), previous miscarriages (5), previous gravidity (6), leisure time physical activity (7), Income (8), smoking (9), previous preterm/low birthweight newborn (10), newborn’s gender (11), education (12), employment (13), dietary supplements (14), folic acid supplements (15), Fe supplements (16), Parity (17), maternal height (18), socioeconomic status (19) and cohabitant status (20), race (21), marital status (22), pre-pregnancy obesity (23), the rate of prenatal steroid administrations (24), polycystic ovary syndrome (25), vomiting (26), sedentary time(27), alcohol (28), insurance coverage (29), sleep durations (30), having a planned pregnancy (31), maternal place of birth (32), enrolment site (33), weight gain during pregnancy (34)

**Supplementary Figure 1. Cochrane risk of bias (graph) for RCTs.**

**
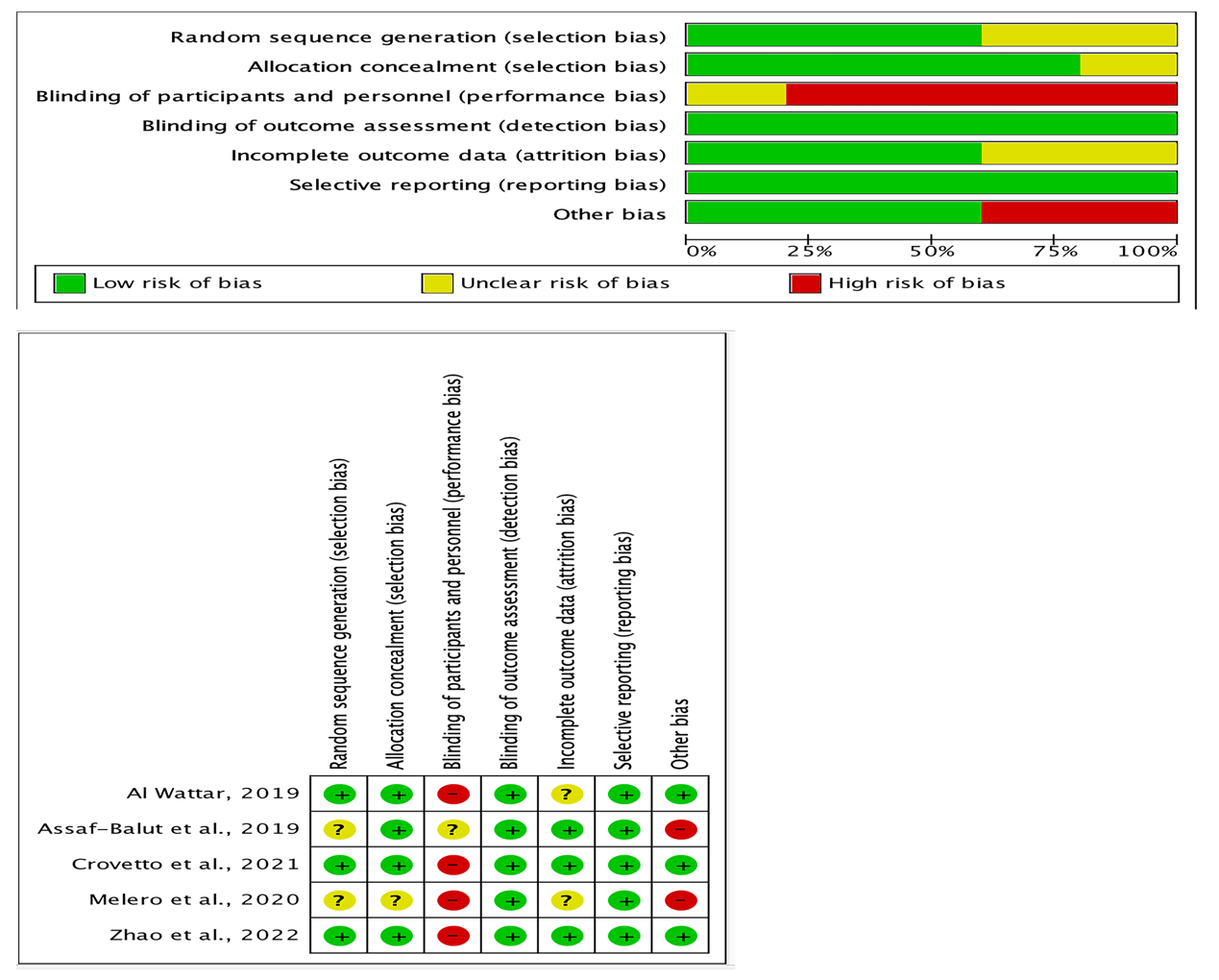
**

**Supplementary Figure 2. Meta-analysis of the maternal Mediterranean dietary and adverse perinatal outcomes (RCTs).**

2. 1Maternal outcomes

2.1.1Gestational diabetes mellitus

**
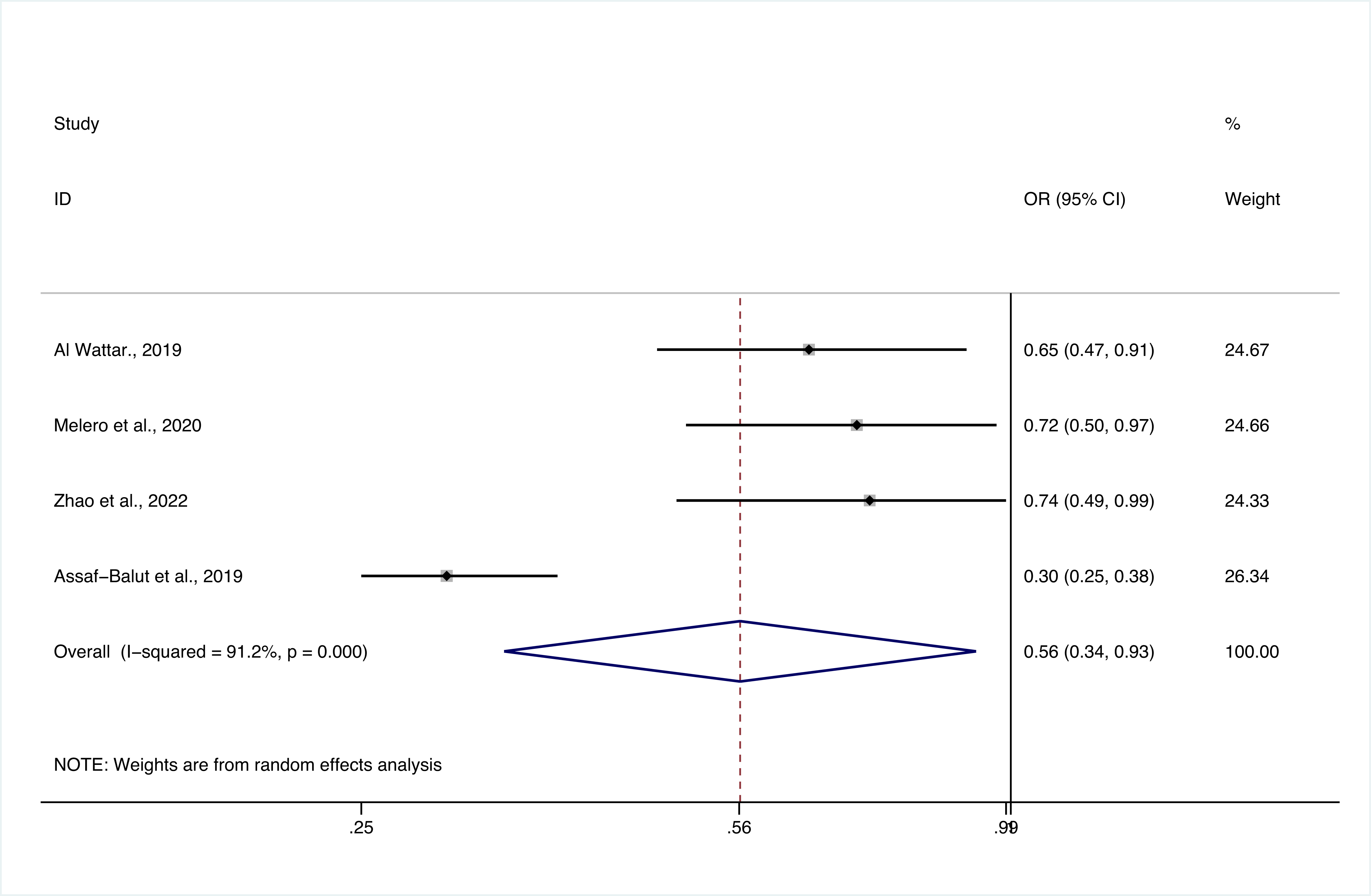
**

2.1.2 Pre-eclampsia


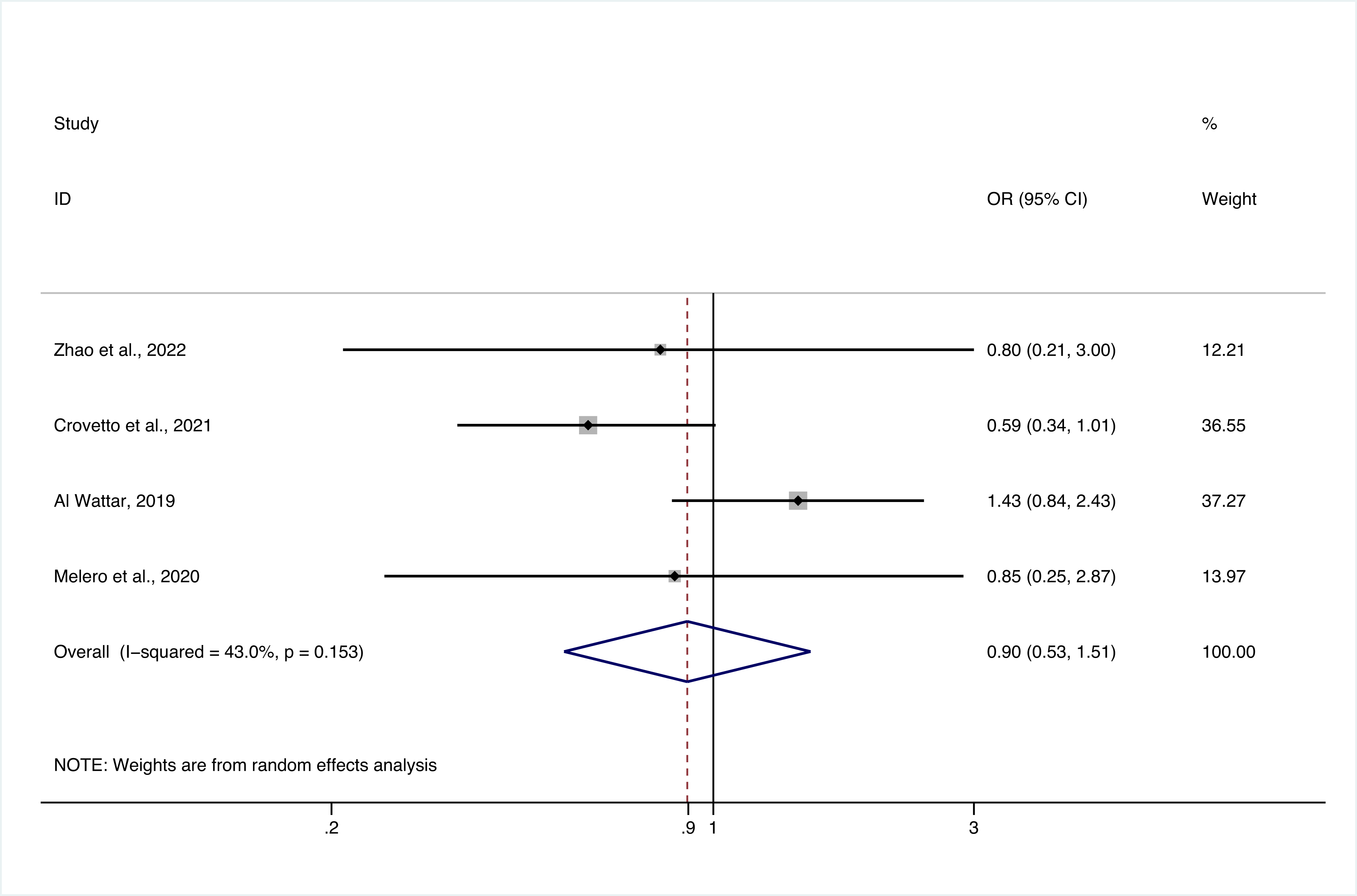


2.1.3 Preterm delivery

**
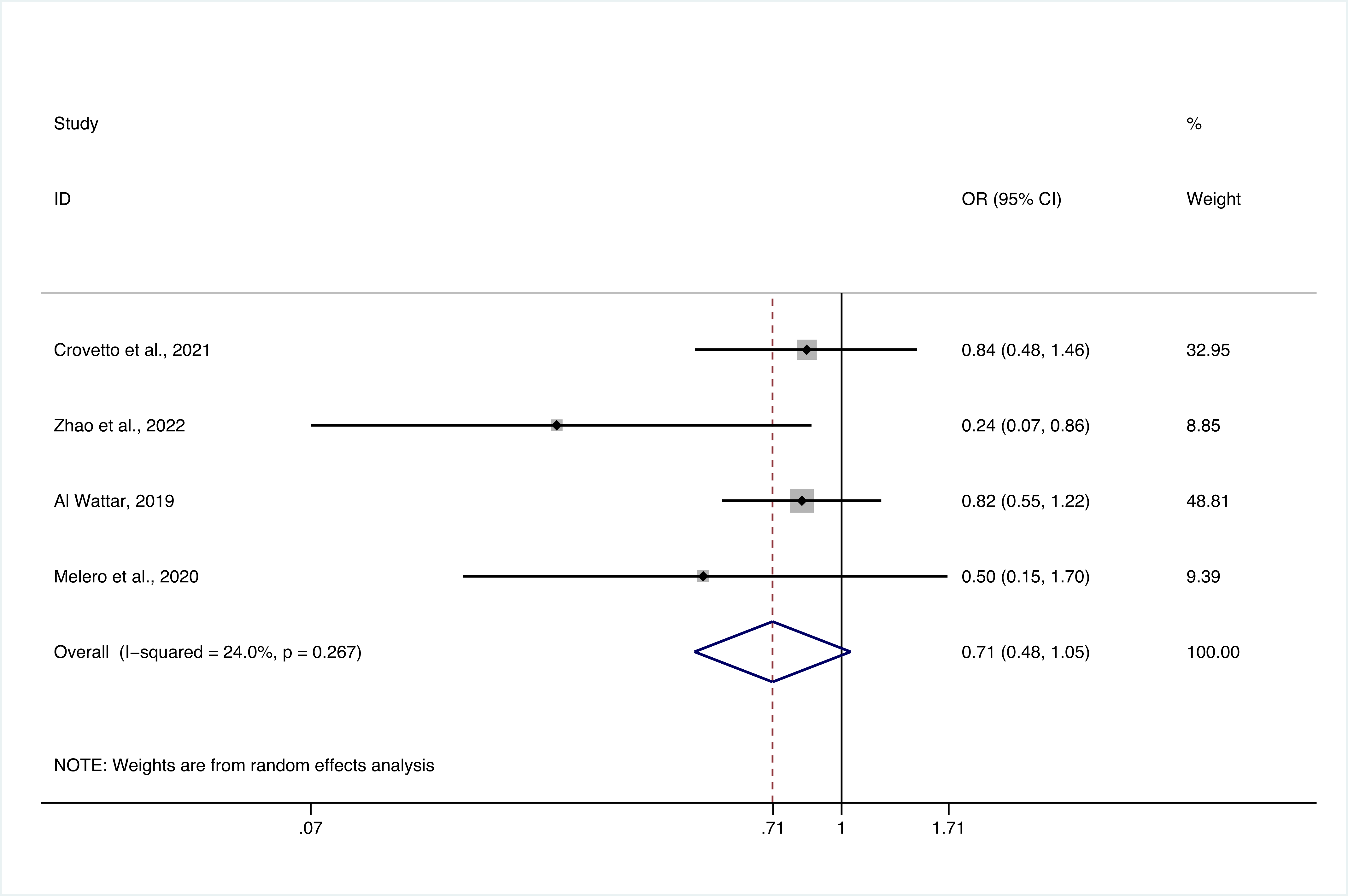
**

2.1.4 Caesarean section

**
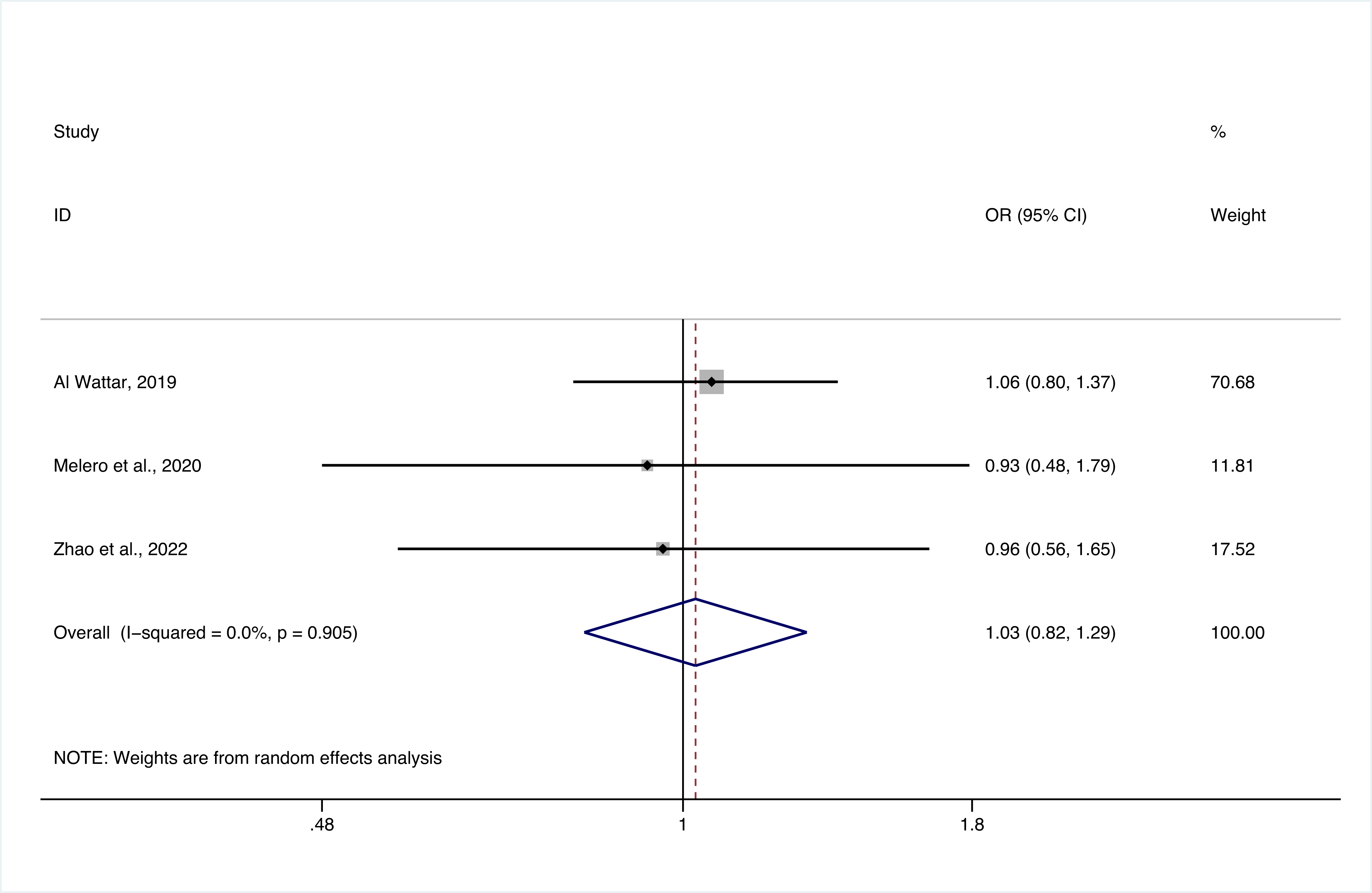
**

2.2 Neonatal outcomes

2.2.1 Small for gestational age

**
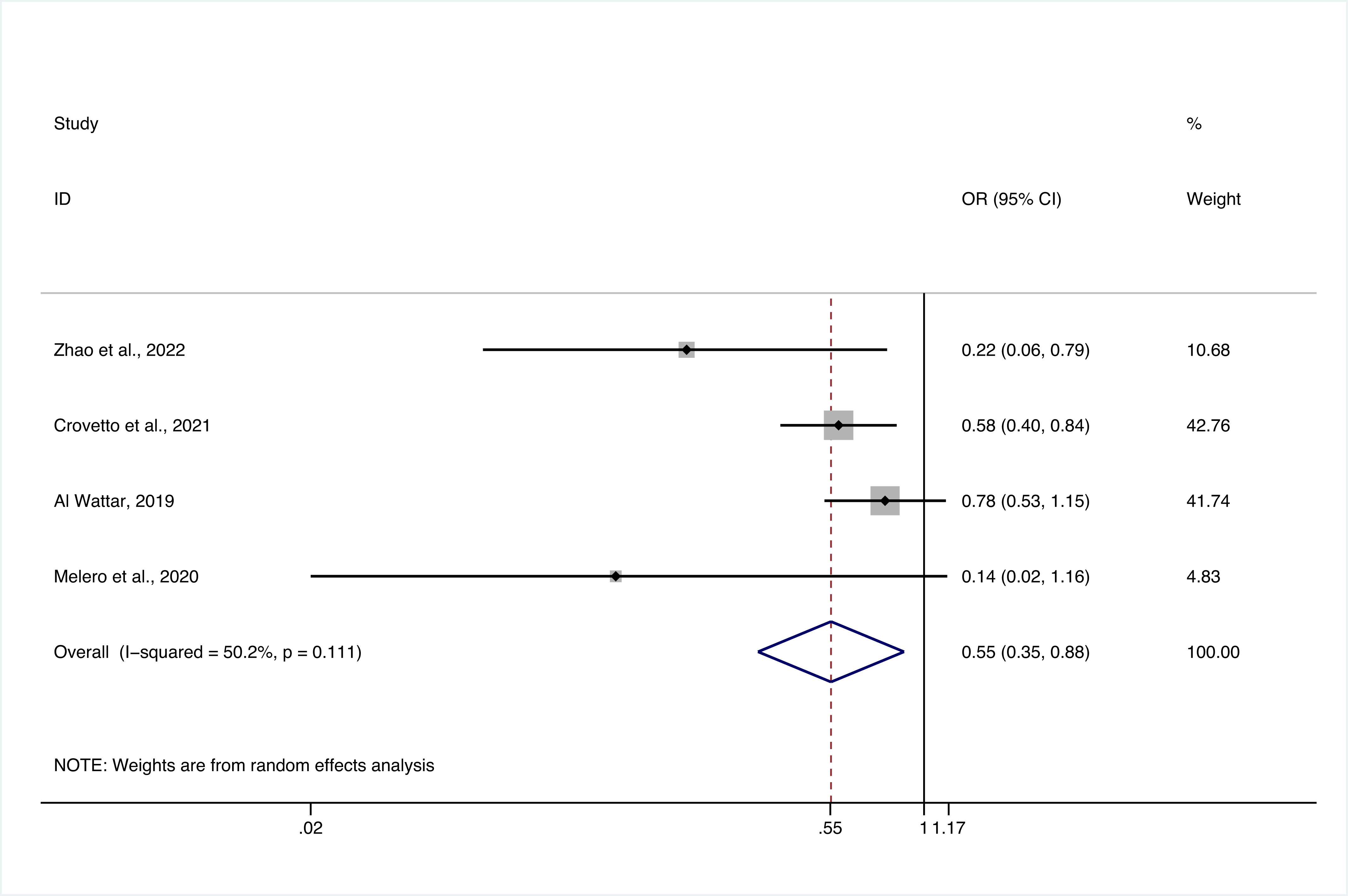
**

- - 1. Large for gestational age

**
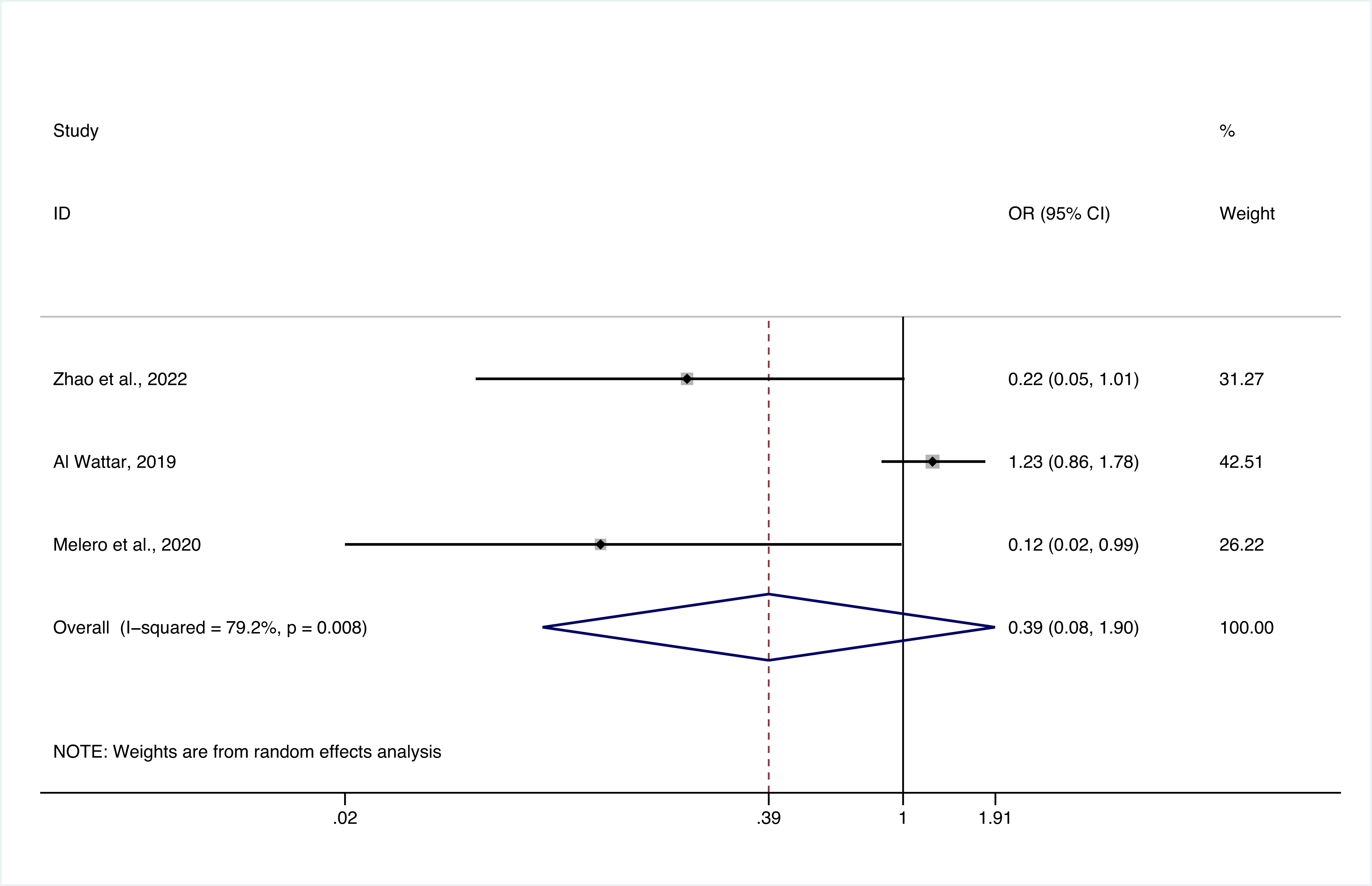
**

- - 1. NICU admission

**
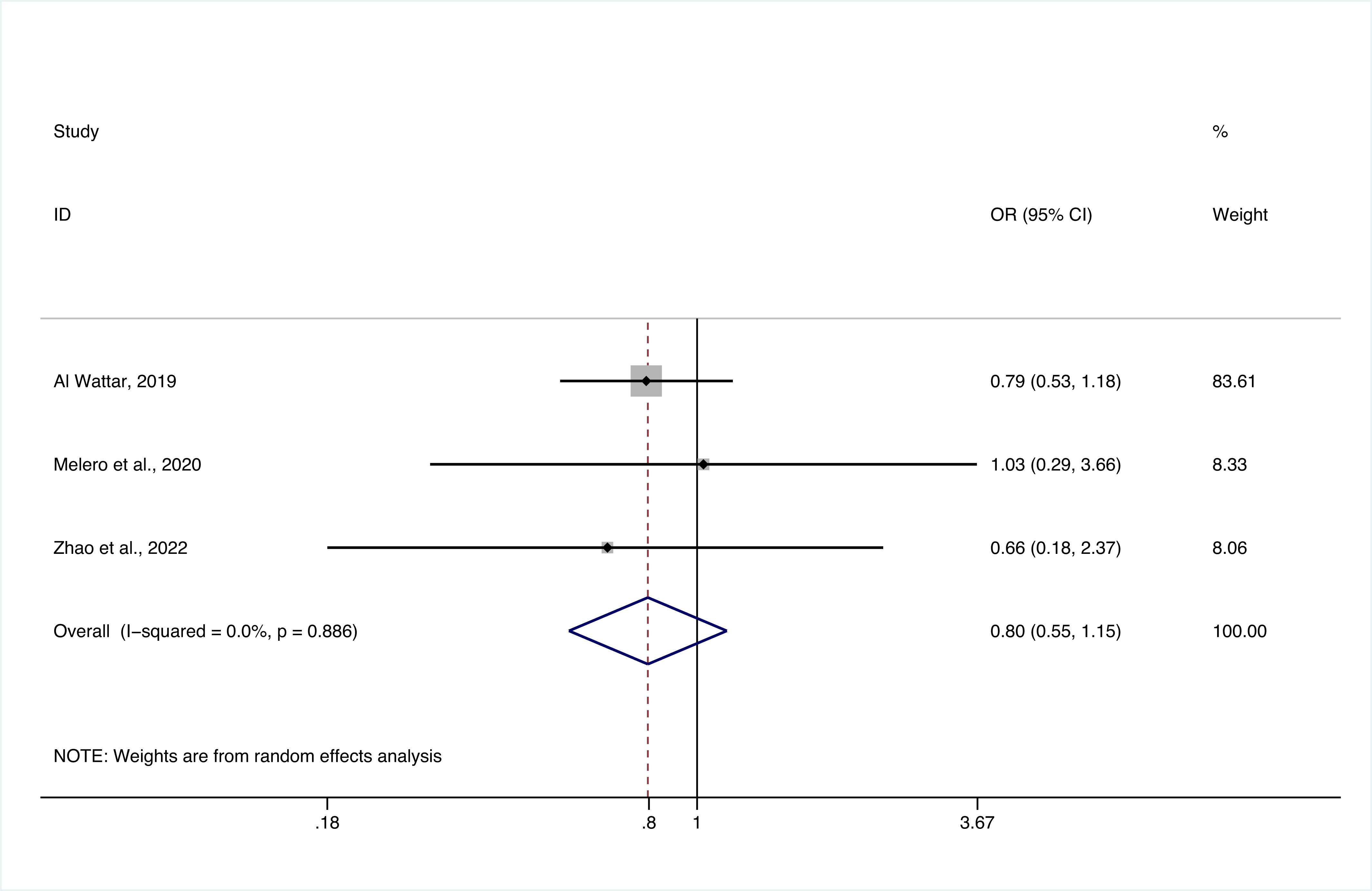
**

2.3 Measured outcomes

2.3.1 Birth weight (g)

**
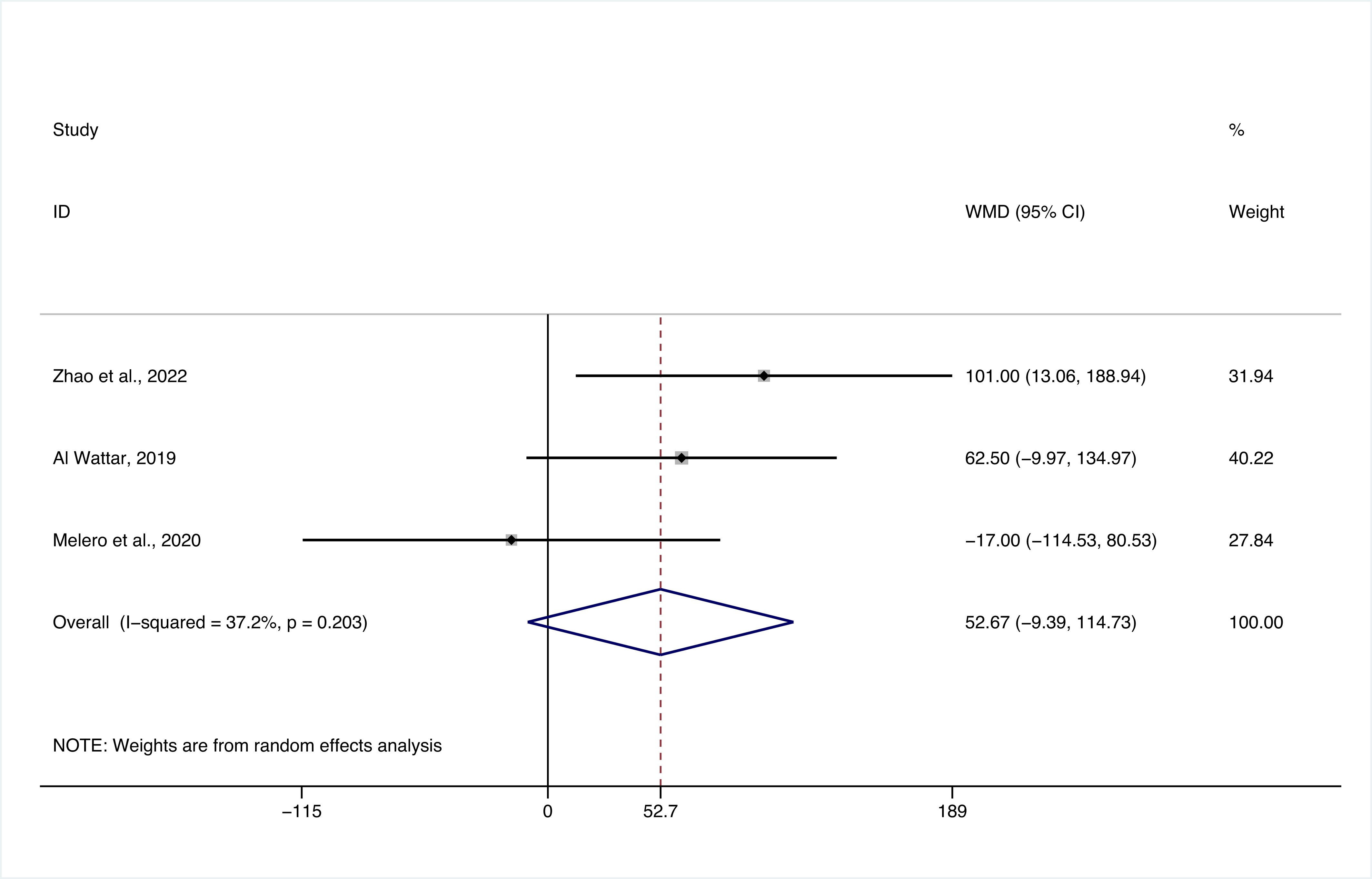
**

**Supplementary Figure 3. Meta-analysis of the maternal Mediterranean dietary and adverse perinatal outcomes (cohort studies).**

- 1. Maternal outcomes

3.1.1 Gestational diabetes mellitus

**
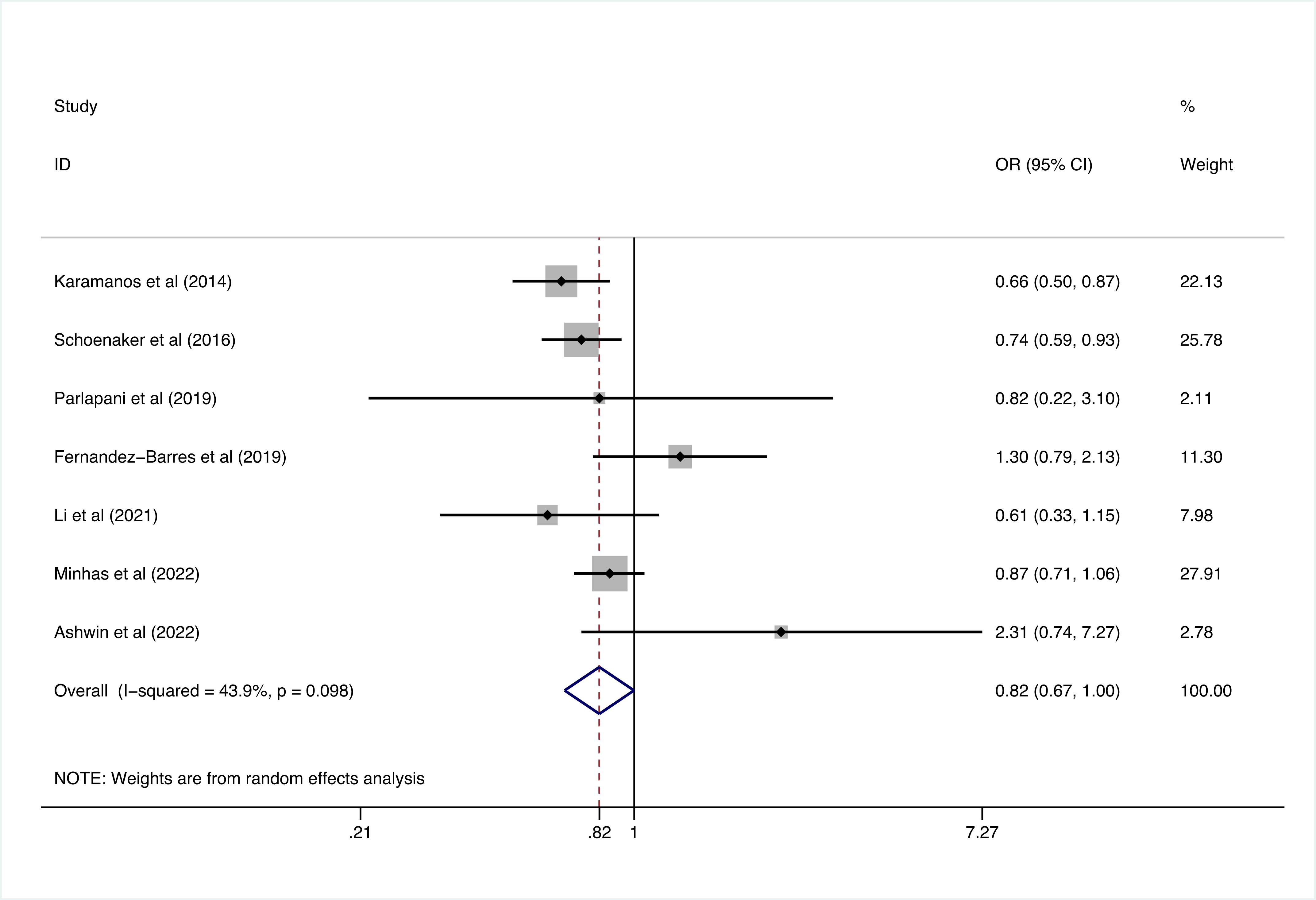
**

3.1.2 Pregnancy-induced hypertension


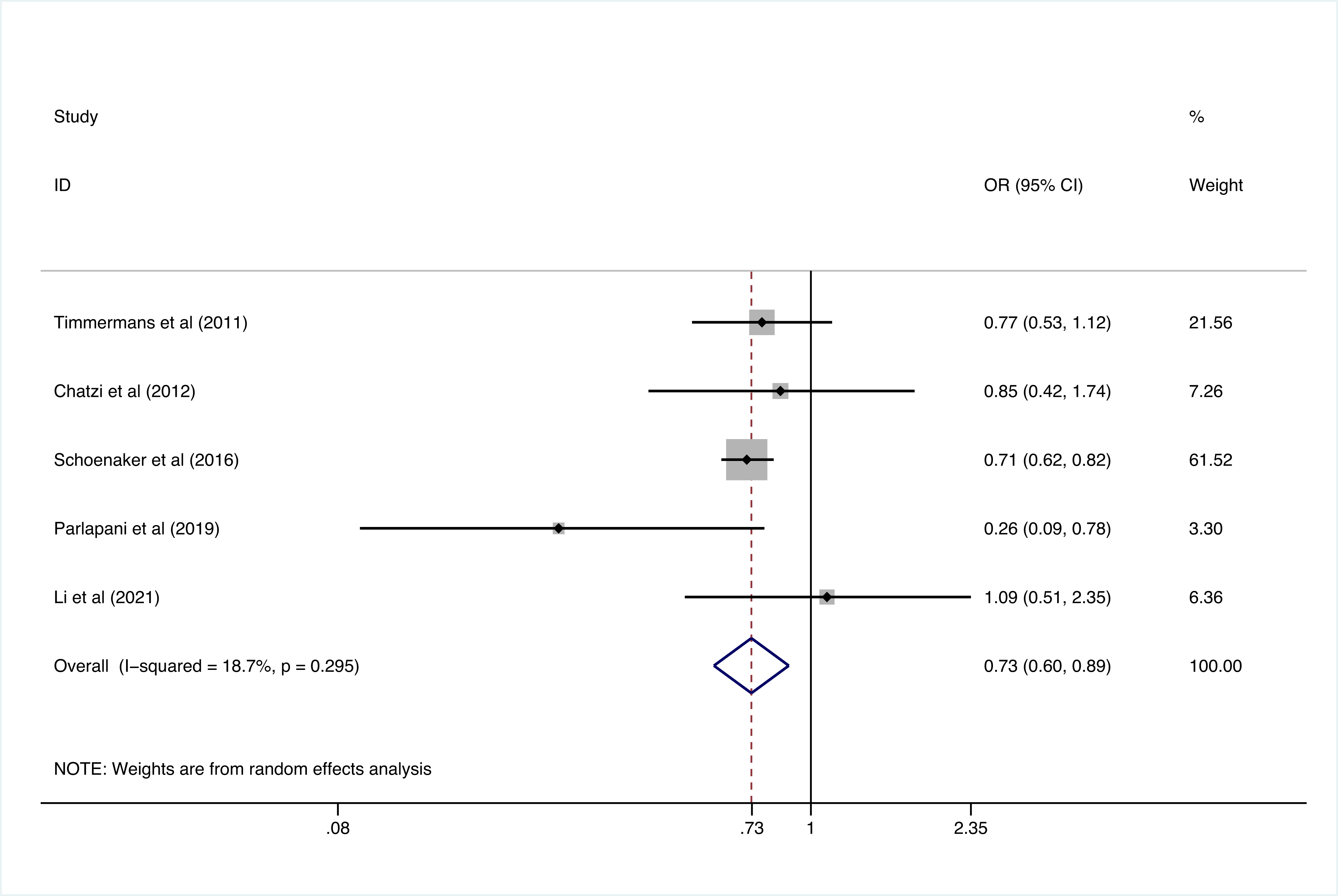
3.1.3 Pre-eclampsia

**
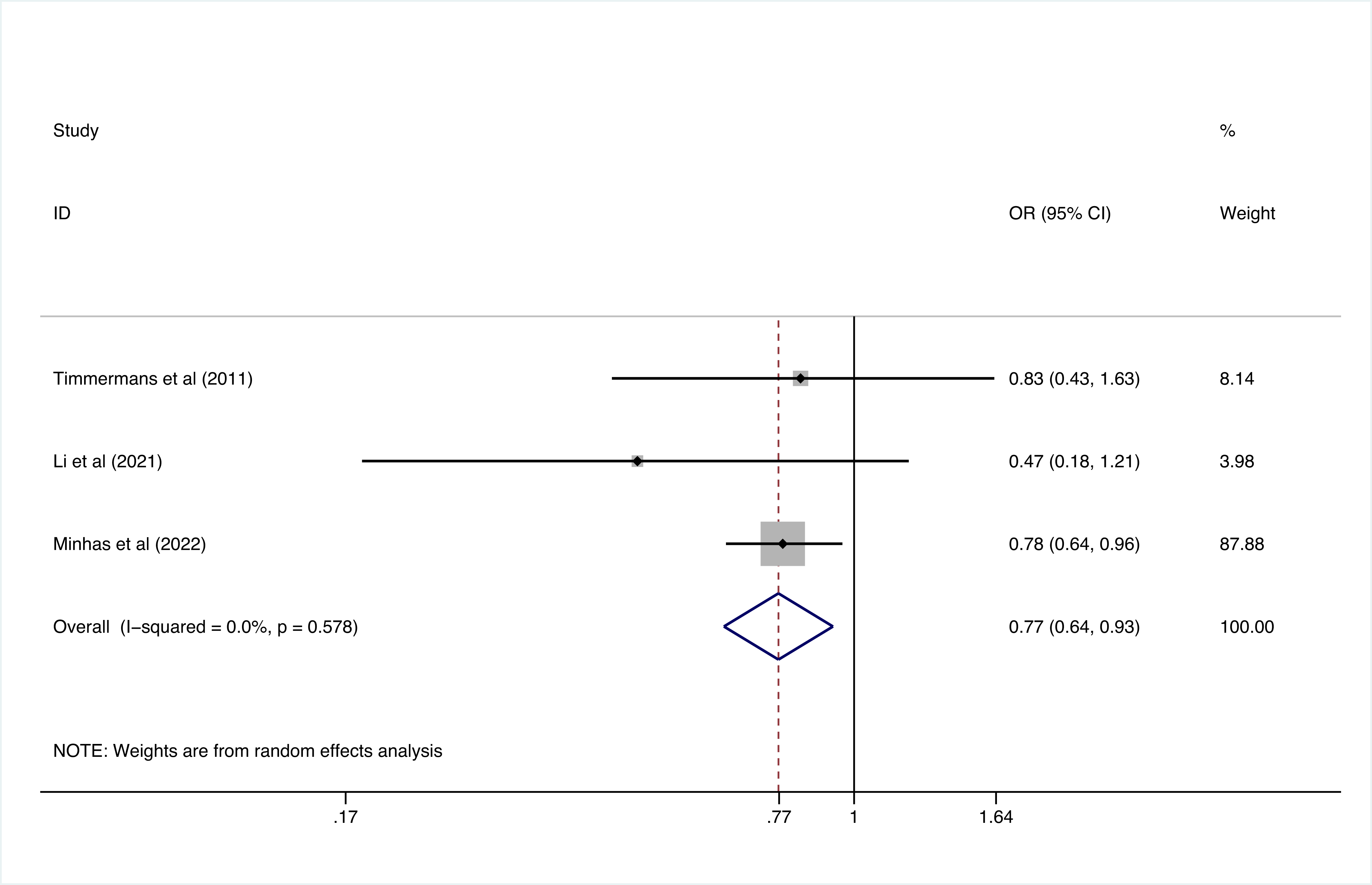
**

3.1.4 Preterm delivery

**
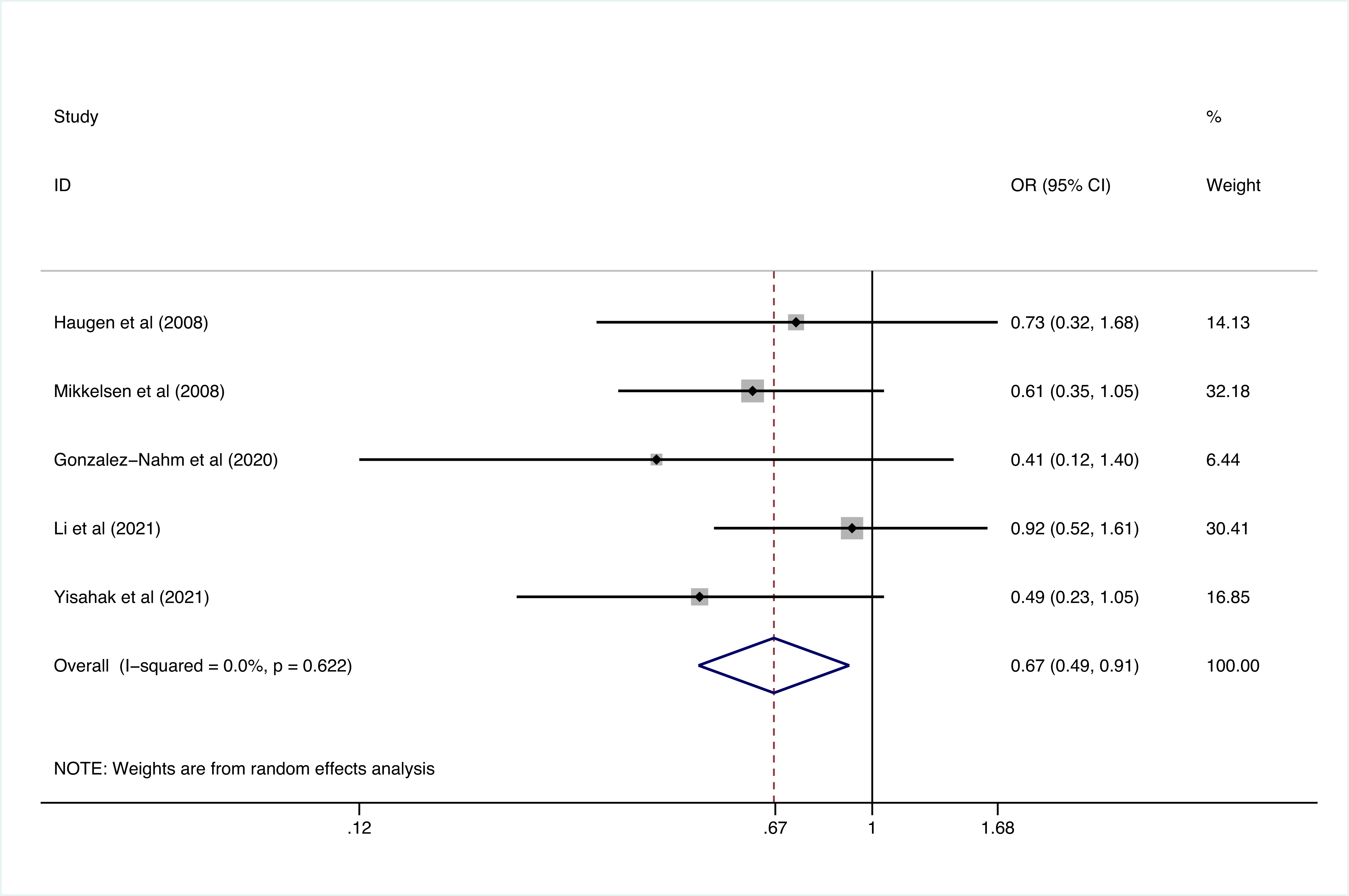
**

3.1.5 Abortion

**
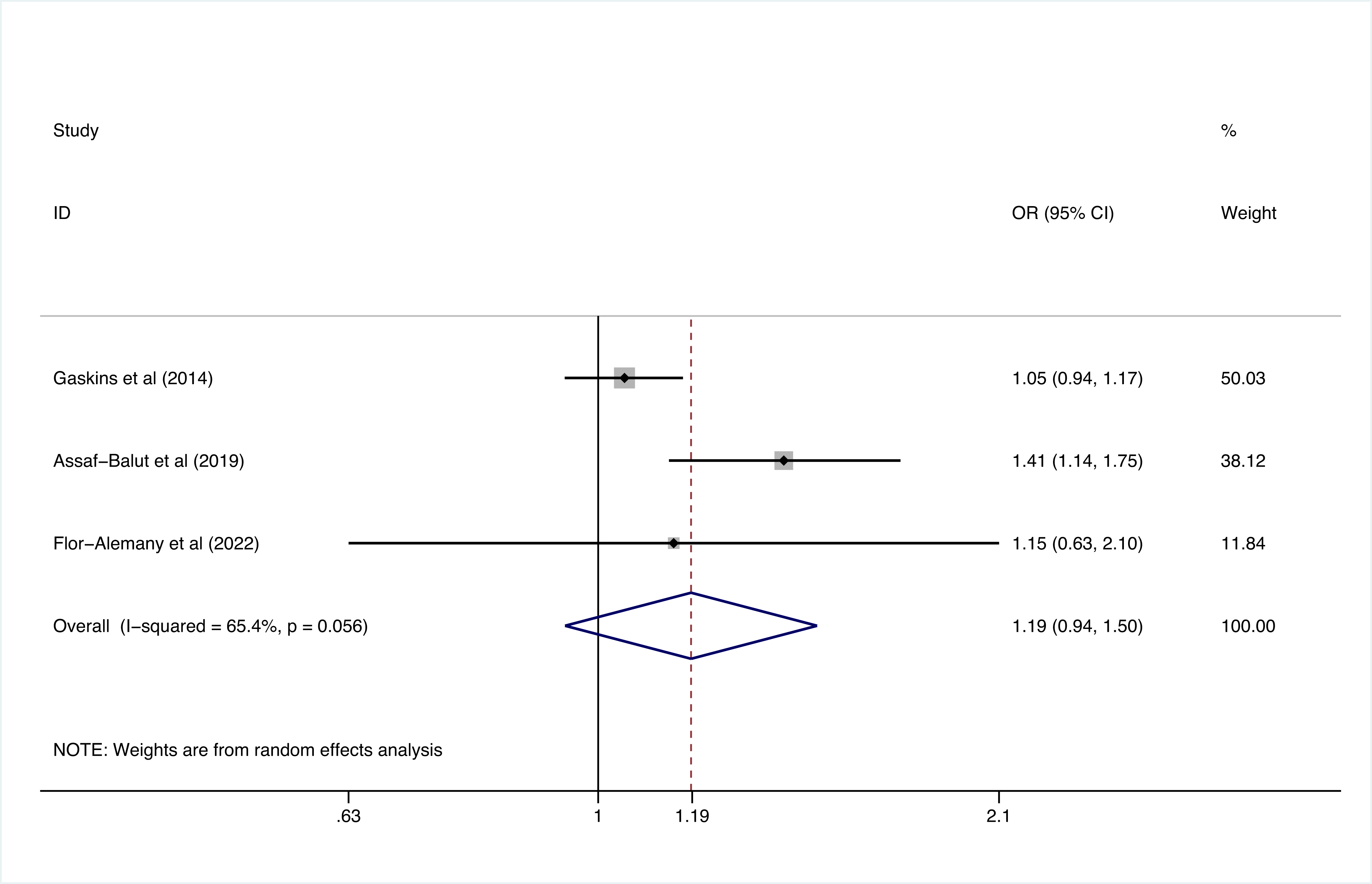
**

3.2 neonatal outcomes

3.2.1 Low birth weight

**
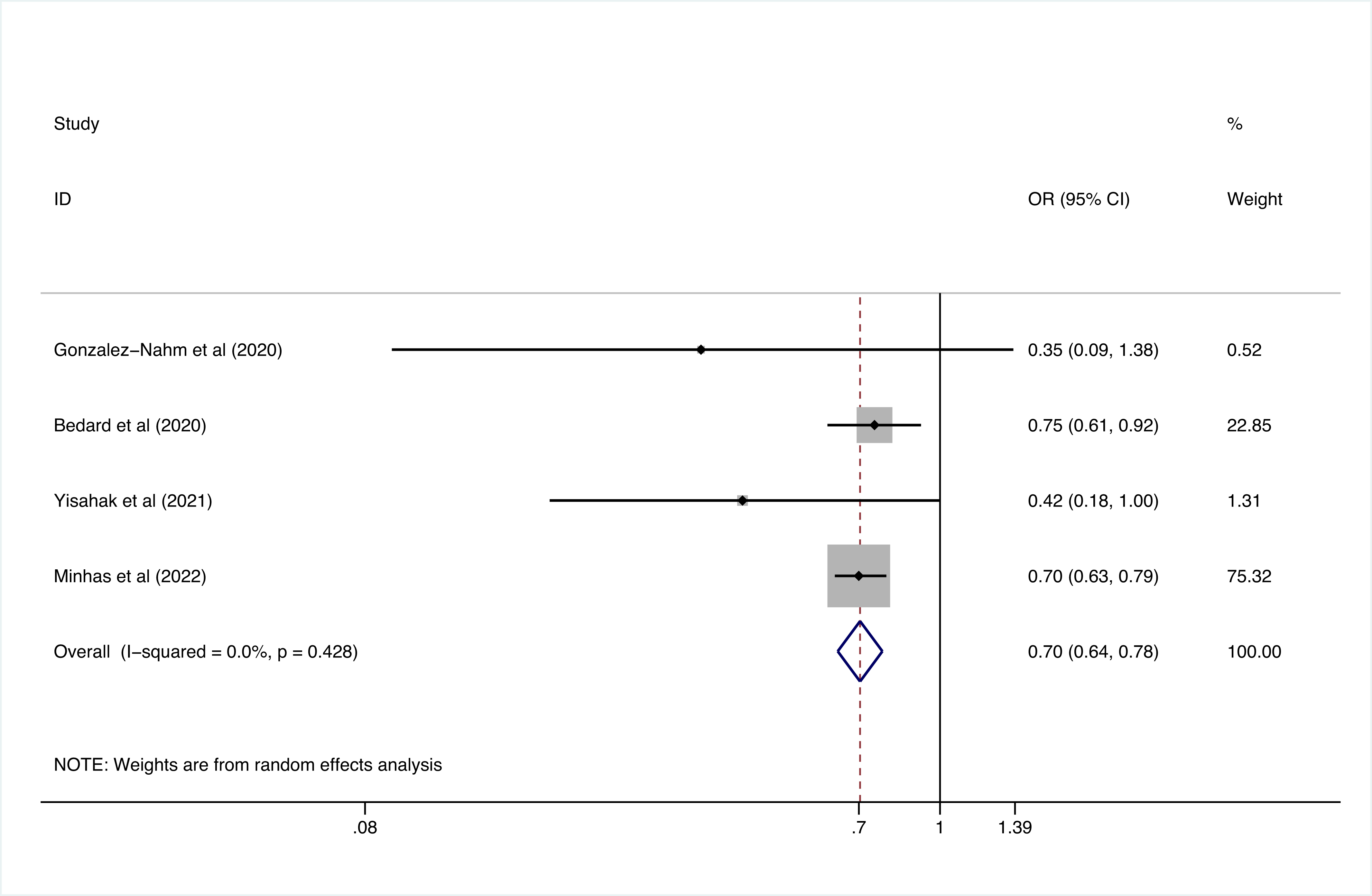
**

3.2.2 Intrauterine growth restriction

**
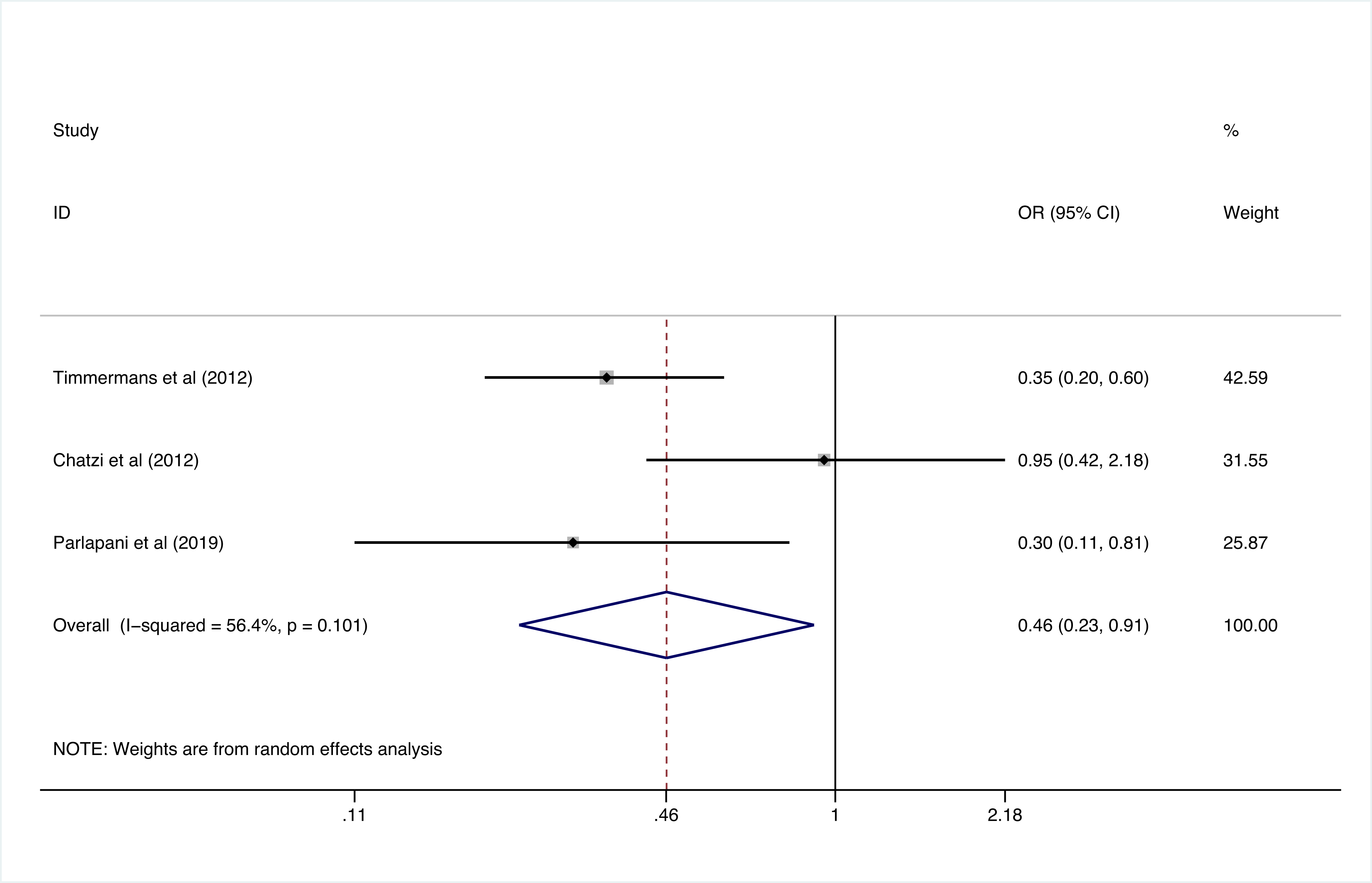
**

3.3 Measured outcomes

3.3.1 Gestational Age at Birth (weeks)

**
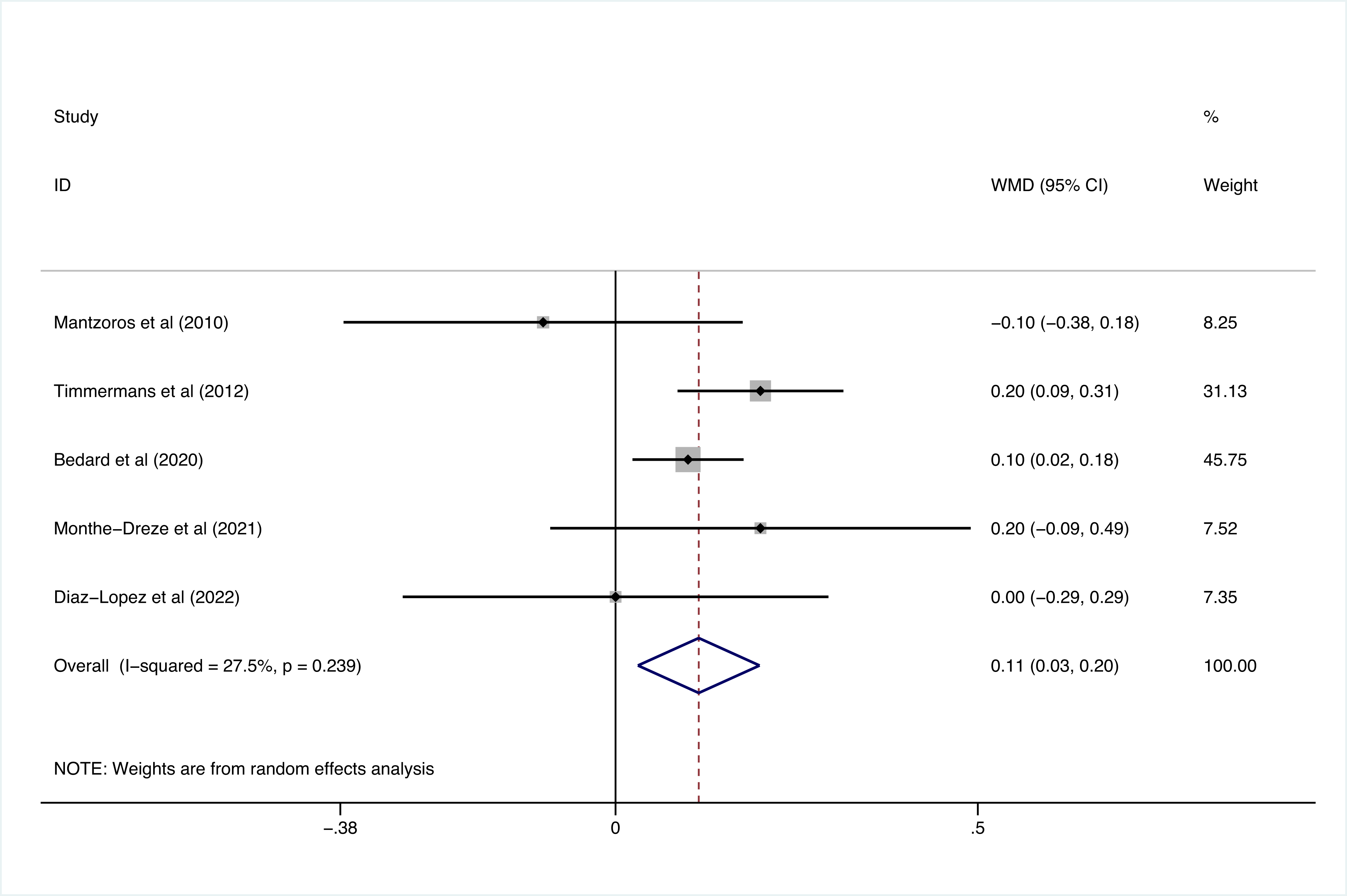
**

3.3.2 Birth weight (g)

**
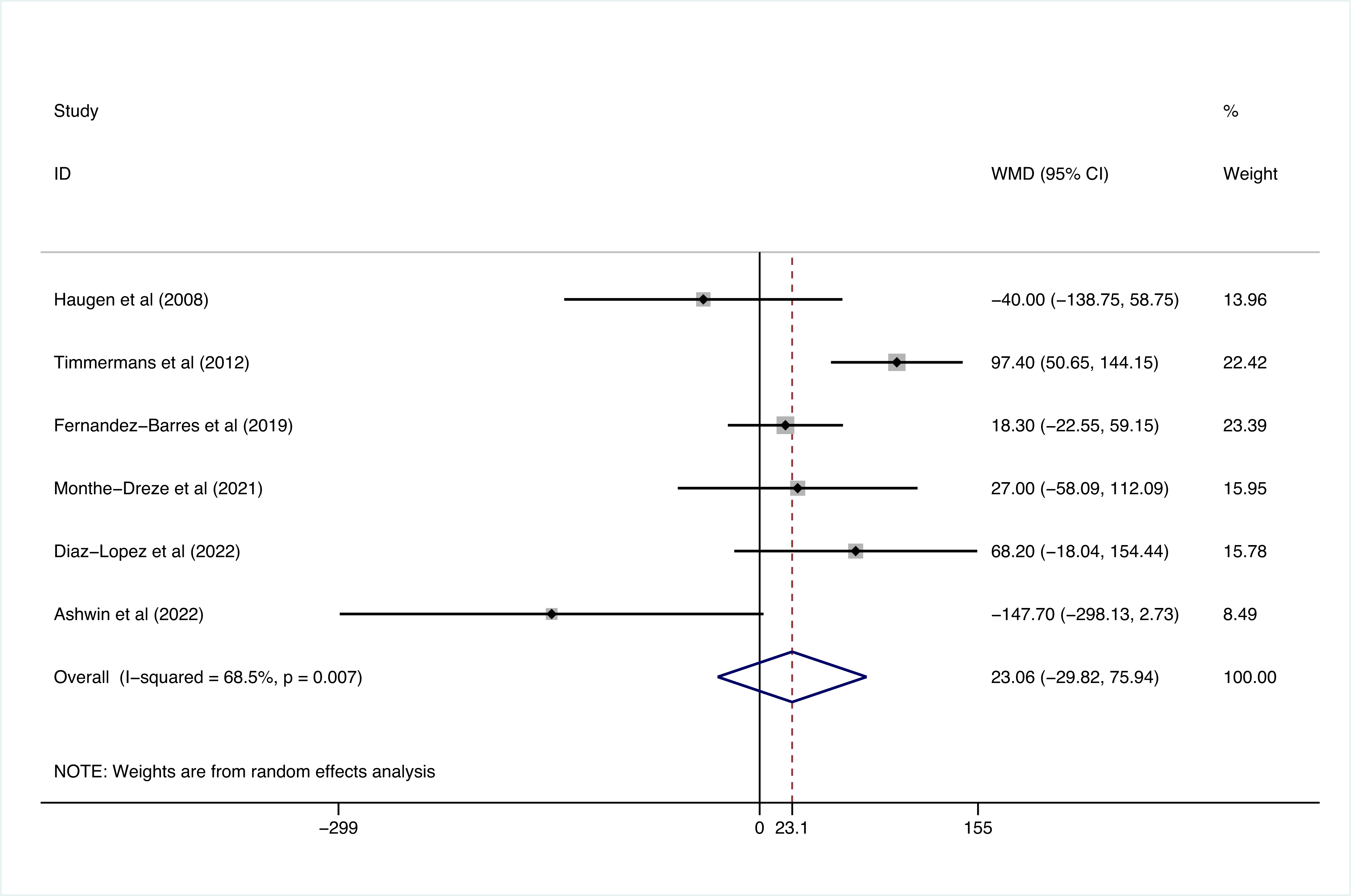
**

**Supplementary Figure 4. Subgroup analysis based on the adjustment of confounders for GDM, pregnancy-induced hypertension, and preterm delivery (cohort studies).**

- 1. Gestational diabetes mellitus


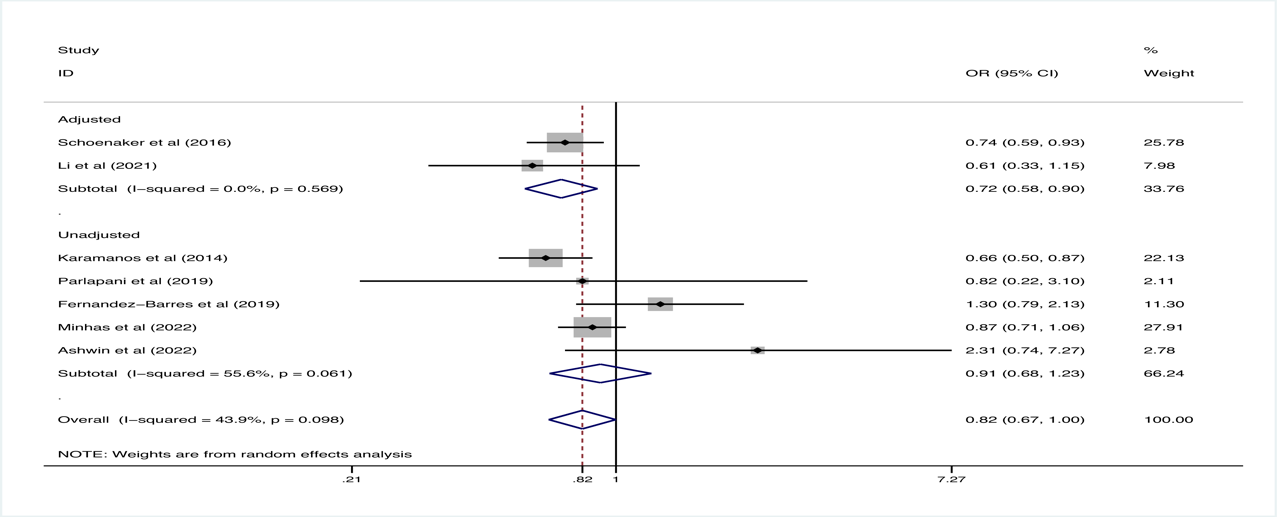


- 1. Pregnancy-induced hypertension


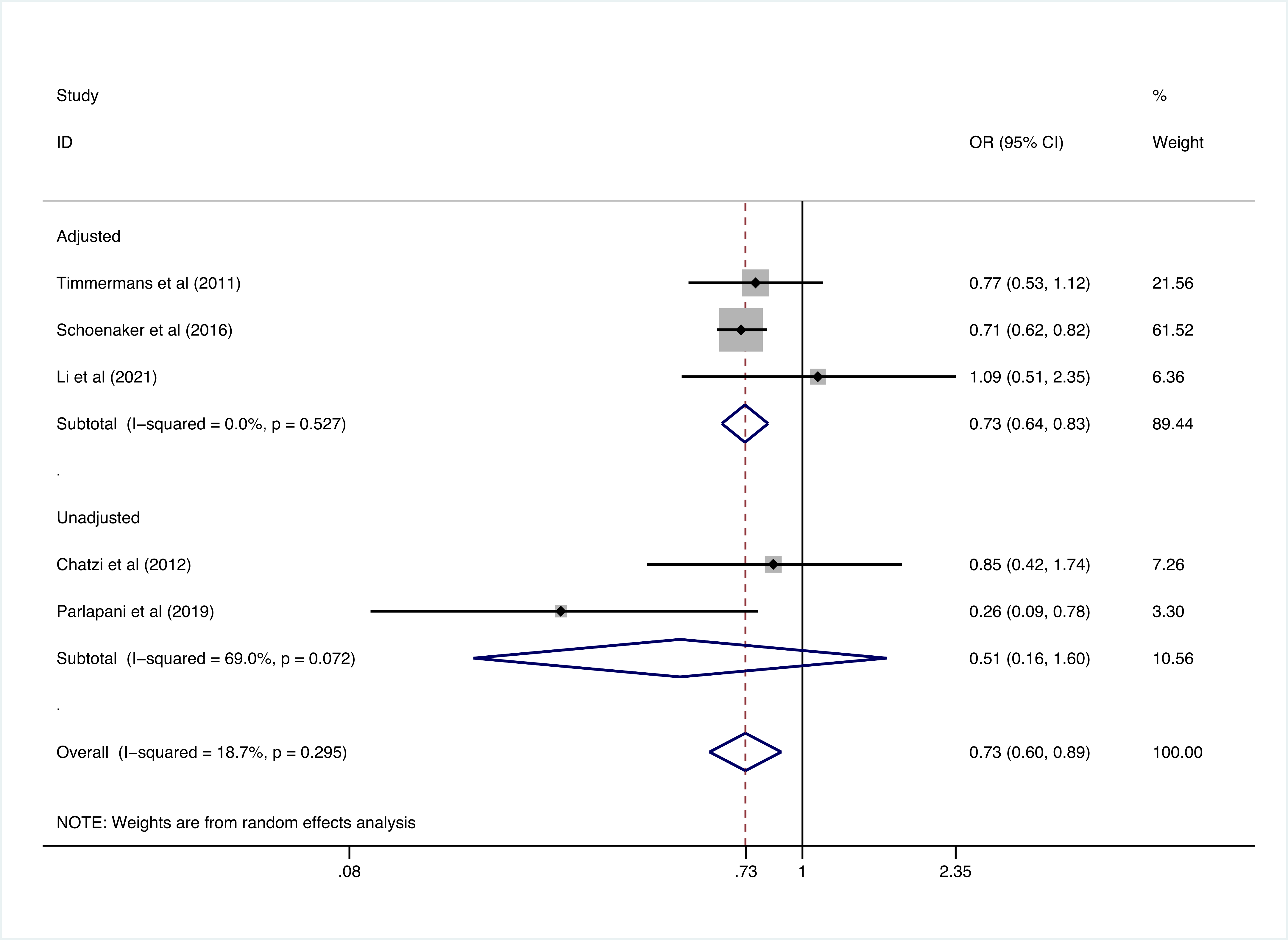


- 1. Preterm delivery

**
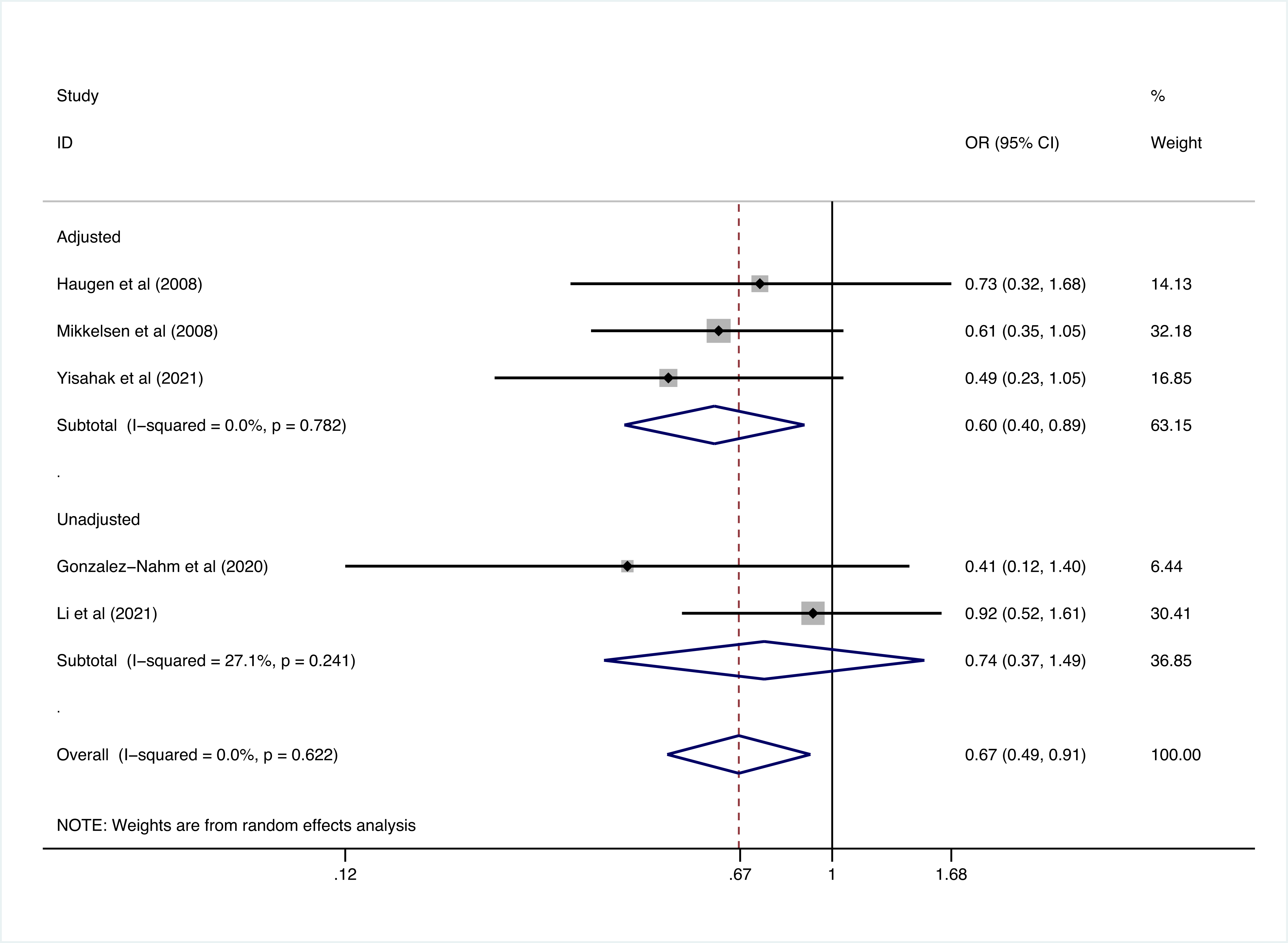
**

**Supplementary Figure 5. Subgroup analysis based on the geographical location of GDM, pregnancy-induced hypertension, preterm delivery, and birth weight (g) (cohort studies).**

5.1 Gestational diabetes mellitus


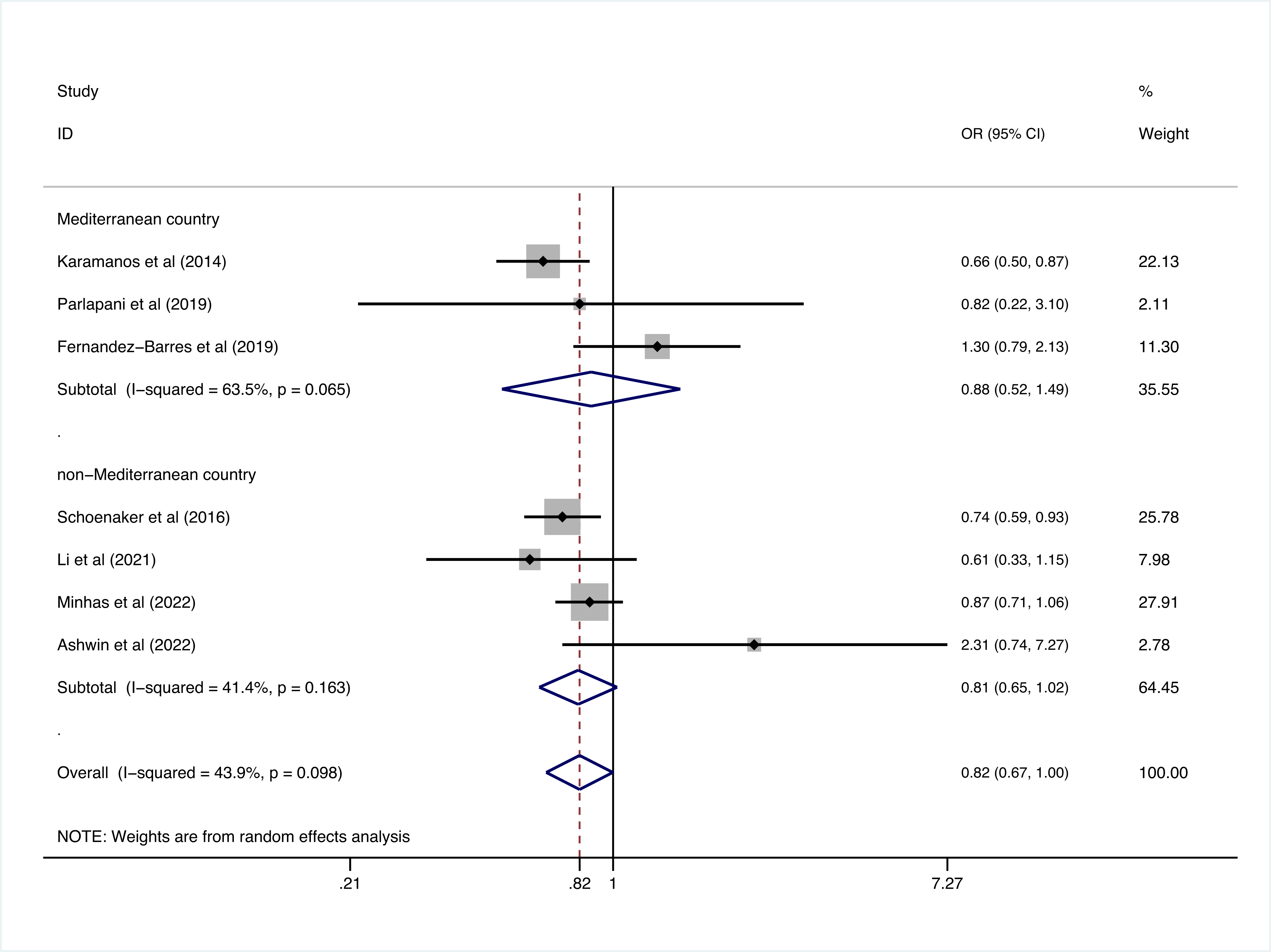
5.2 Pregnancy-induced hypertension


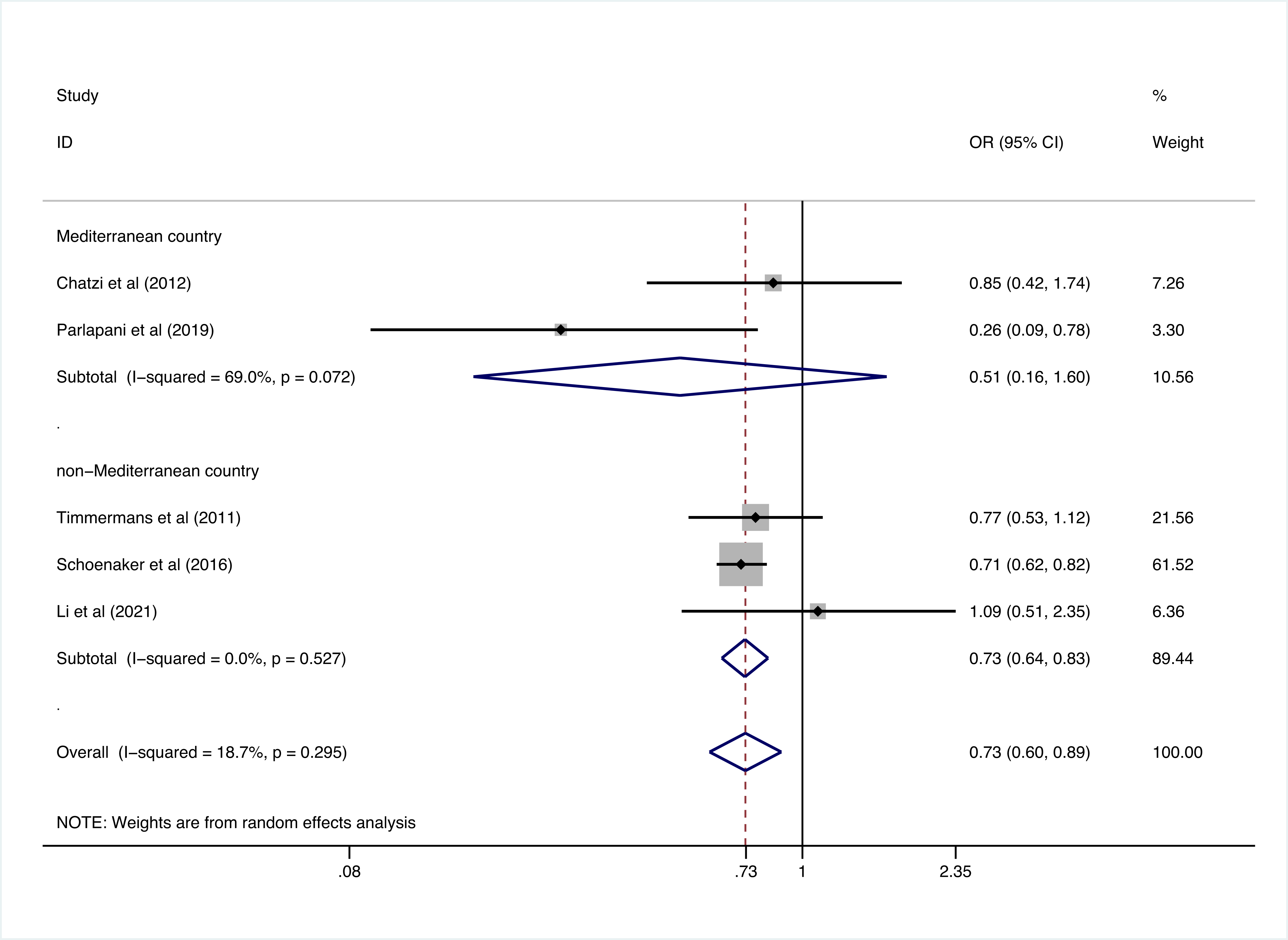


5.3 Birth weight (g)


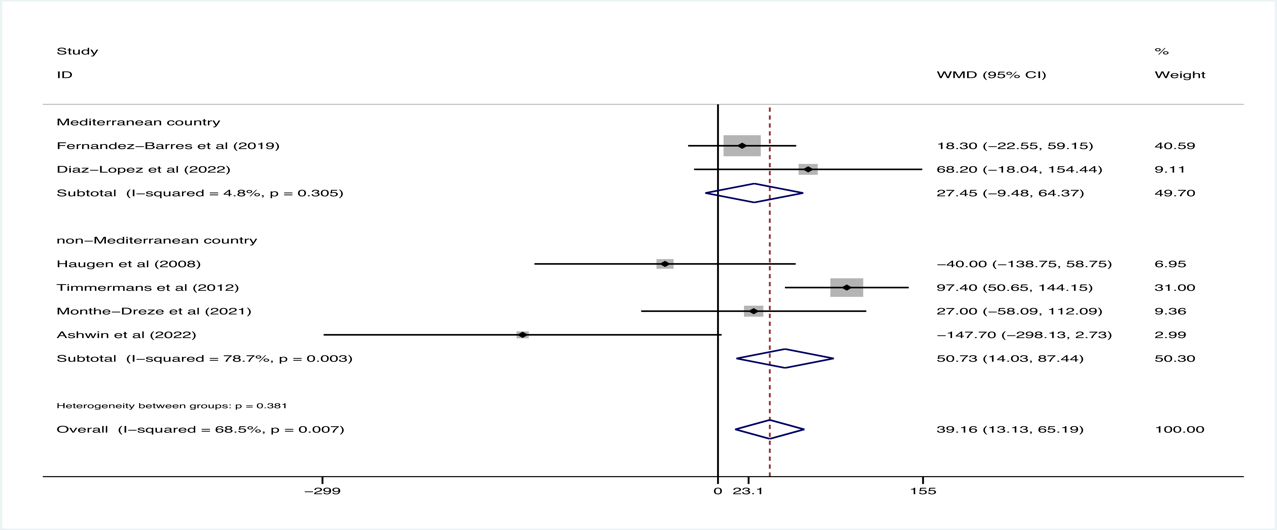


**Supplementary Figure 6. Sensitivity analysis by omitting individual studies (cohort studies).**

6.1 Gestational diabetes mellitus

**
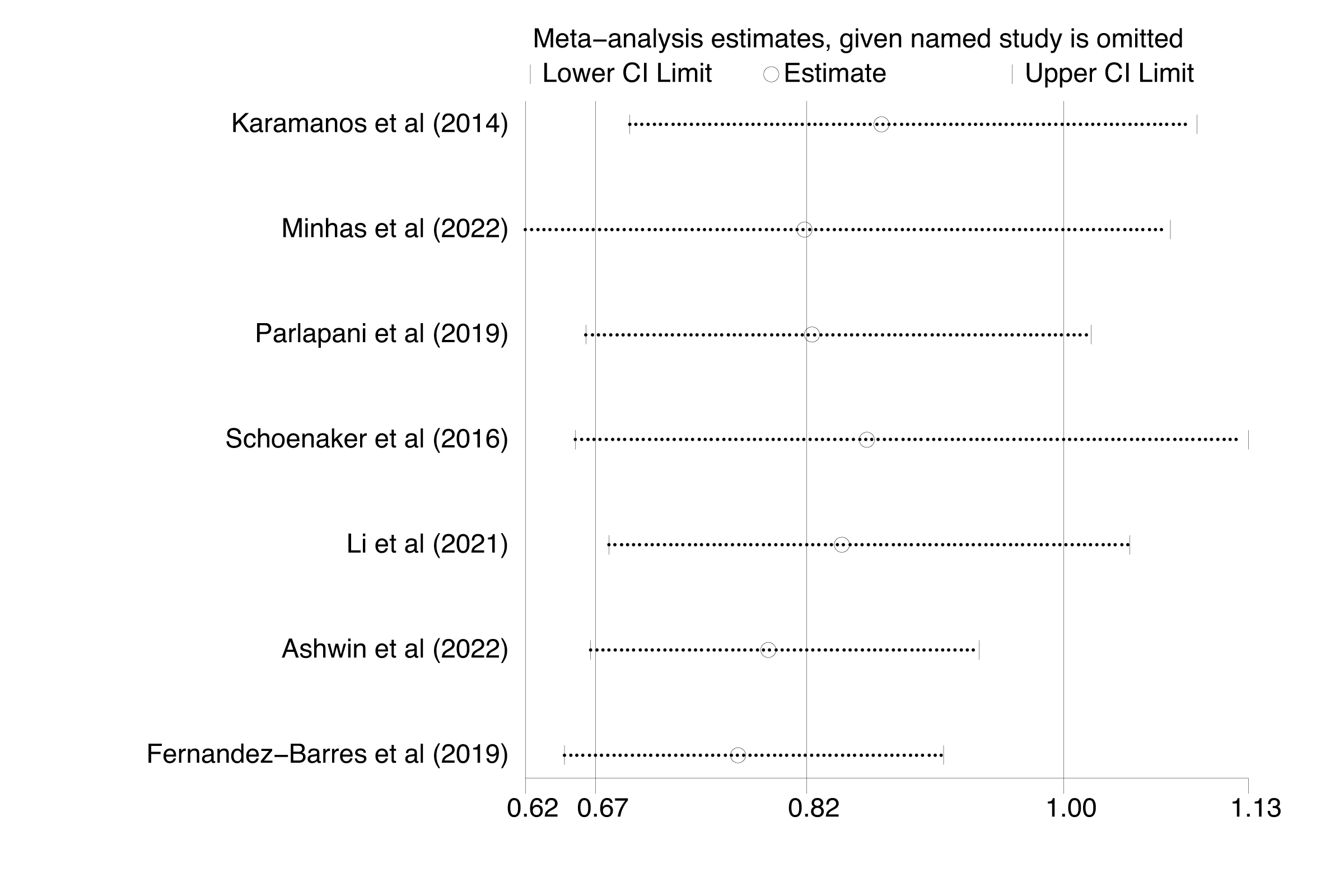
**

6.2 Pregnancy-induced hypertension

**
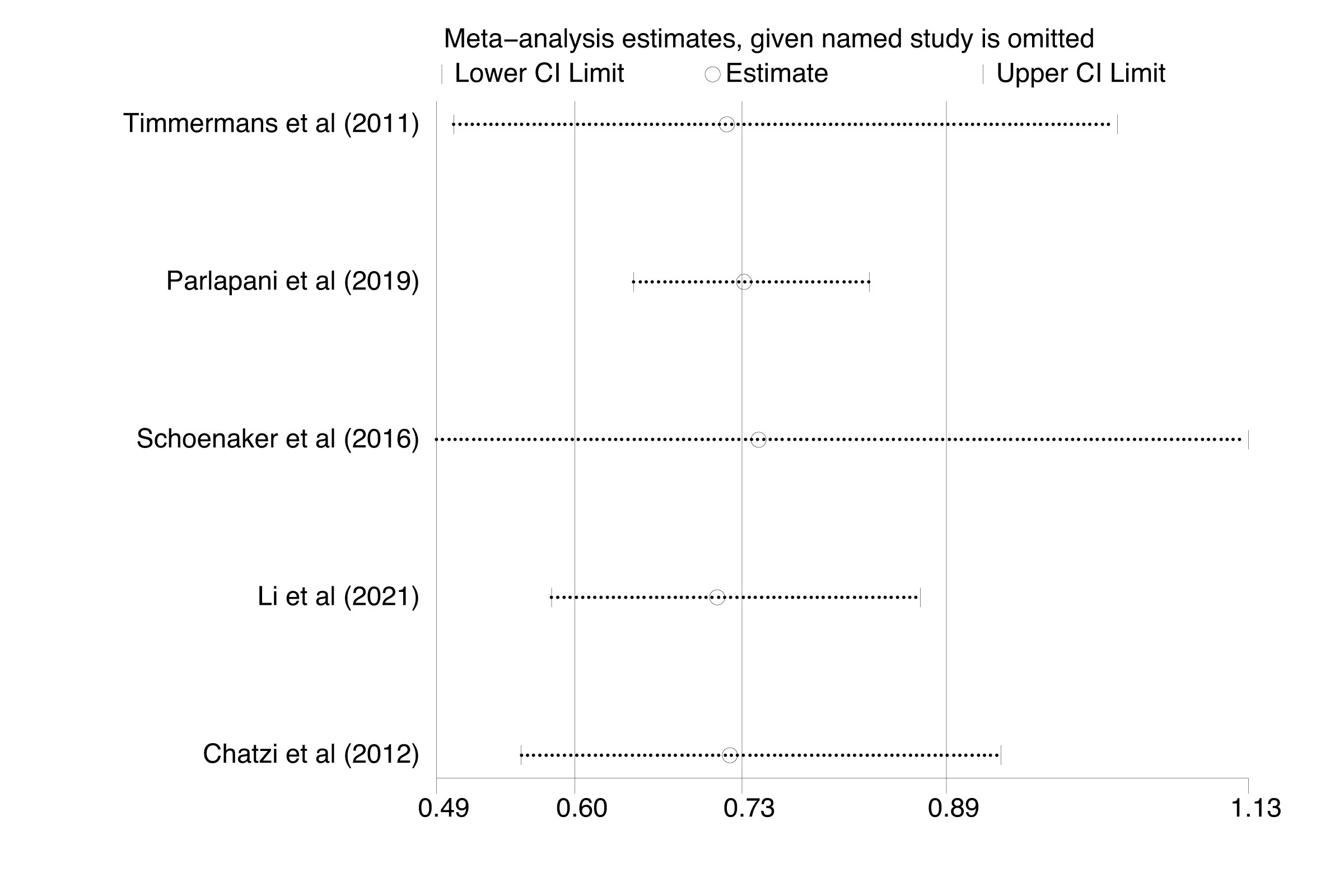
**

6.3 Preterm delivery

**
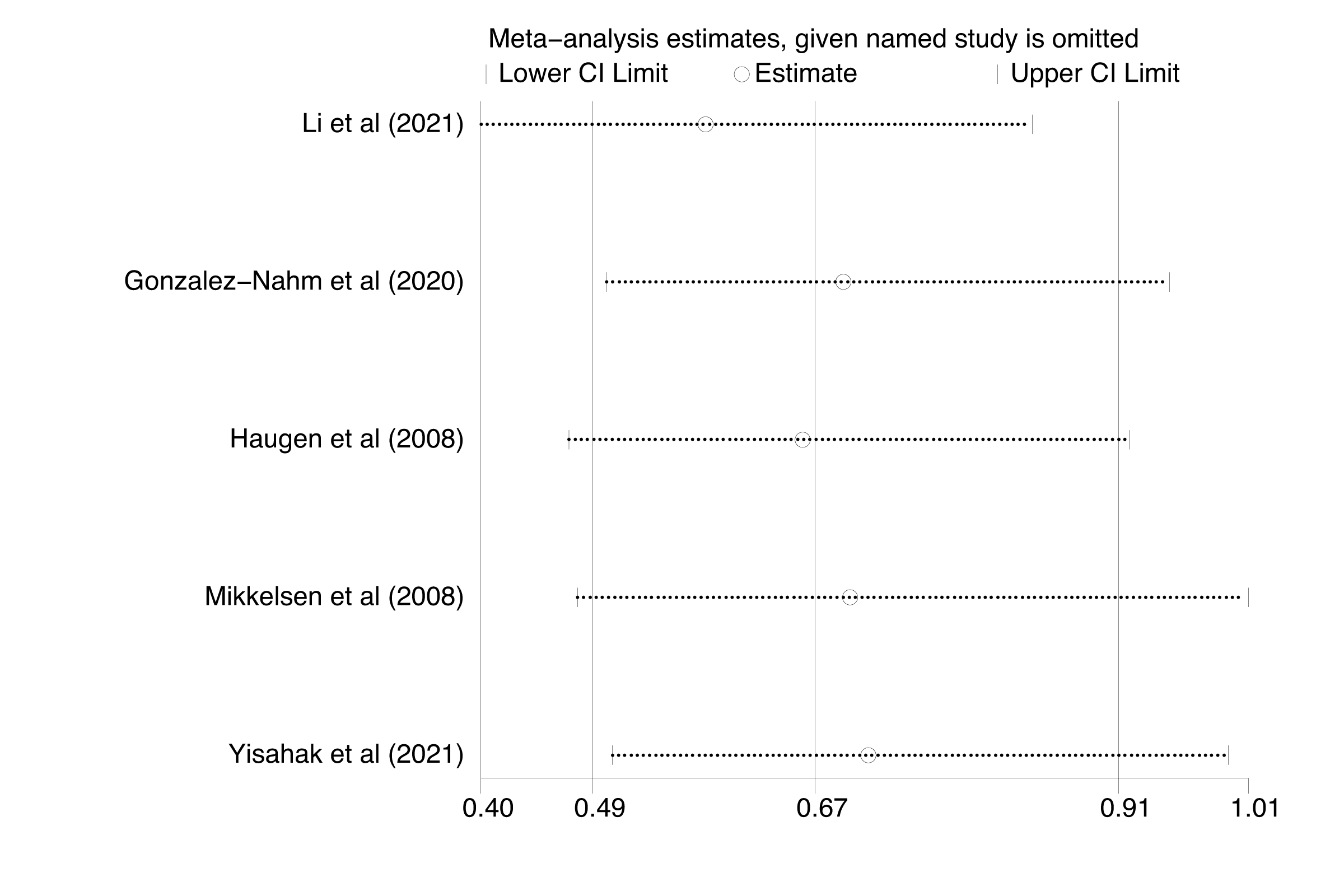
**

6.4 Gestational Age at birth (weeks)

**
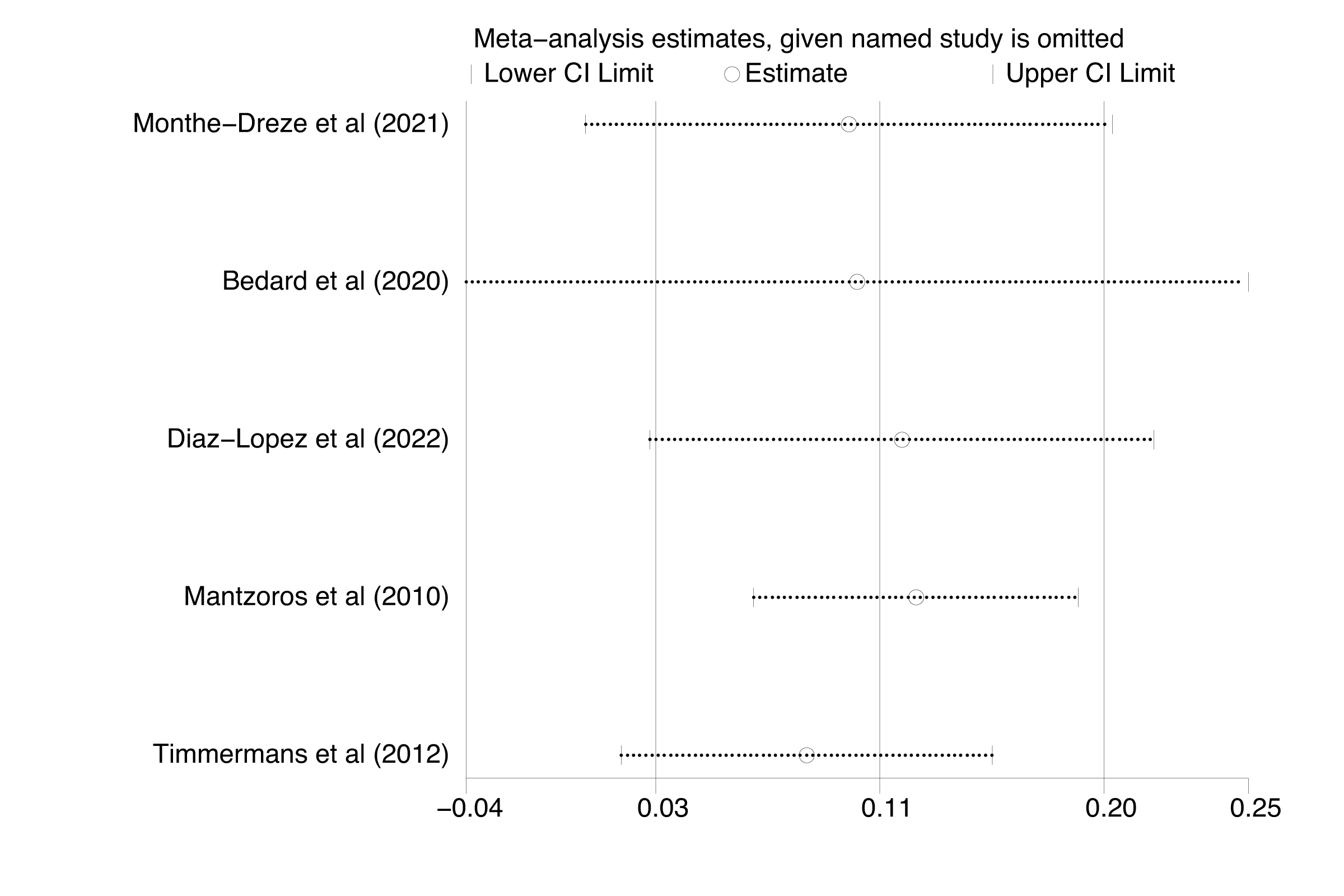
**

6.5 Birth weight (g)

**
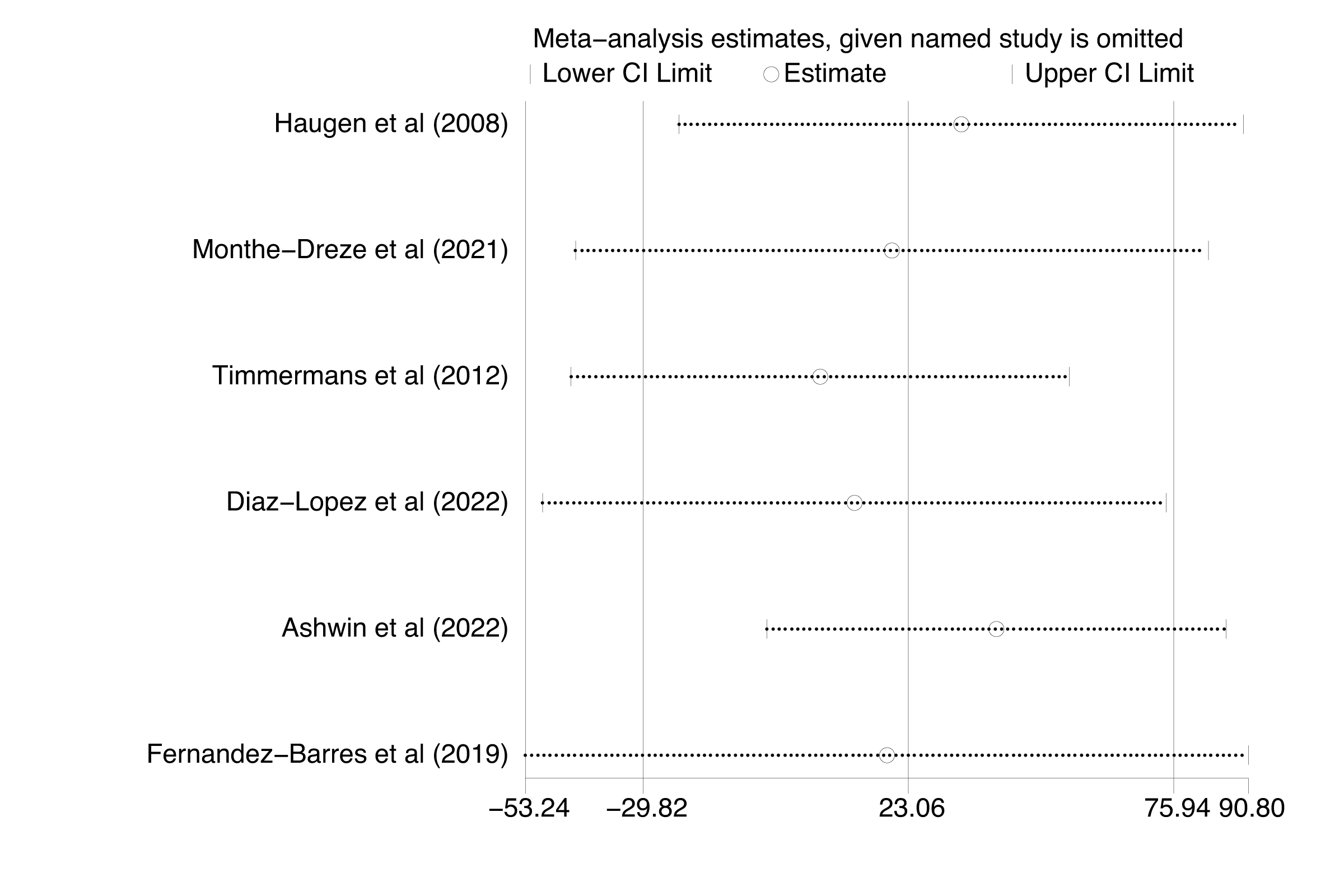
**

**Supplementary Reference:**

1. H Al Wattar B, Dodds J, Placzek A, Beresford L, Spyreli E, Moore A, et al; ESTEEM study group. Mediterranean-style diet in pregnant women with metabolic risk factors (ESTEEM): A pragmatic multicenter randomized trial. PLoS Med. 2019 Jul 23;16(7):e1002857.
2. Crovetto F, Crispi F, Casas R, Martín-Asuero A, Borràs R, Vieta E, et al. IMPACT BCN Trial Investigators. Effects of Mediterranean Diet or Mindfulness-Based Stress Reduction on Prevention of Small-for-Gestational Age Birth Weights in Newborns Born to At-Risk Pregnant Individuals: The IMPACT BCN Randomized Clinical Trial. JAMA. 2021 Dec 7;326(21):2150-2160.
3. Melero V, García de la Torre N, Assaf-Balut C, Jiménez I, Del Valle L, Durán A, et al. Effect of a Mediterranean Diet-Based Nutritional Intervention on the Risk of Developing Gestational Diabetes Mellitus and Other Maternal-Fetal Adverse Events in Hispanic Women Residents in Spain. Nutrients. 2020 Nov 14;12(11):3505.
4. Zhao L, Zhang P, Zheng Q, Deka A, Choudhury R, Rastogi S. Does a MediDiet With Additional Extra Virgin Olive Oil and Pistachios Reduce the Incidence of Gestational Diabetes? Endocr Pract. 2022 Feb;28(2):135-141.
5. Assaf-Balut C, Garcia de la Torre N, Durán A, Bordiu E, Del Valle L, Familiar C, et al. An Early, Universal Mediterranean Diet-Based Intervention in Pregnancy Reduces Cardiovascular Risk Factors in the "Fourth Trimester". J Clin Med. 2019 Sep 19;8(9):1499.
6. Chatzi L, Mendez M, Garcia R, Roumeliotaki T, Ibarluzea J, Tardón A, et al. INMA and RHEA study groups. Mediterranean diet adherence during pregnancy and fetal growth: INMA (Spain) and RHEA (Greece) mother-child cohort studies. Br J Nutr. 2012 Jan;107(1):135-45.
7. Gonzalez-Nahm S, Nihlani K, S House J, L Maguire R, G Skinner H, Hoyo C. Associations between Maternal Cadmium Exposure with Risk of Preterm Birth and Low after Birth Weight Effect of Mediterranean Diet Adherence on Affected Prenatal Outcomes. Toxics. 2020 Oct 20;8(4):90.
8. Haugen M, Meltzer HM, Brantsaeter AL, Mikkelsen T, Osterdal ML, Alexander J, et al. Mediterranean-type diet and risk of preterm birth among women in the Norwegian Mother and Child Cohort Study (MoBa): a prospective cohort study. Acta Obstet Gynecol Scand. 2008;87(3):319-24.
9. Mikkelsen TB, Osterdal ML, Knudsen VK, Haugen M, Meltzer HM, Bakketeig L, et al. Association between a Mediterranean-type diet and risk of preterm birth among Danish women: a prospective cohort study. Acta Obstet Gynecol Scand. 2008;87(3):325-30.
10. Minhas AS, Hong X, Wang G, Rhee DK, Liu T, Zhang M, et al. Mediterranean-Style Diet and Risk of Preeclampsia by Race in the Boston Birth Cohort. J Am Heart Assoc. 2022 May 3;11(9):e022589.
11. Monthé-Drèze C, Rifas-Shiman SL, Aris IM, Shivappa N, Hebert JR, Sen S, et al. Maternal diet in pregnancy is associated with differences in child body mass index trajectories from birth to adolescence. Am J Clin Nutr. 2021 Apr 6;113(4):895-904.
12. Parlapani E, Agakidis C, Karagiozoglou-Lampoudi T, Sarafidis K, Agakidou E, Athanasiadis A, et al. The Mediterranean diet adherence by pregnant women delivering prematurely: association with size at birth and complications of prematurity. J Matern Fetal Neonatal Med. 2019 Apr;32(7):1084-1091.
13. Schoenaker DA, Soedamah-Muthu SS, Mishra GD. Quantifying the mediating effect of body mass index on the relation between a Mediterranean diet and development of maternal pregnancy complications: the Australian Longitudinal Study on Women's Health. Am J Clin Nutr. 2016 Sep;104(3):638-45.
14. Timmermans S, Steegers-Theunissen RP, Vujkovic M, Bakker R, den Breeijen H, Raat H, et al. Major dietary patterns and blood pressure patterns during pregnancy: the Generation R Study. Am J Obstet Gynecol. 2011 Oct;205(4):337.e1-12.
15. Timmermans S, Steegers-Theunissen RP, Vujkovic M, den Breeijen H, Russcher H, Lindemans J, et al. The Mediterranean diet and fetal size parameters: the Generation R Study. Br J Nutr. 2012 Oct 28;108(8):1399-409.
16. Yisahak SF, Mumford SL, Grewal J, Li M, Zhang C, Grantz KL, et al. Maternal diet patterns during early pregnancy in relation to neonatal outcomes. Am J Clin Nutr. 2021 Jul 1;114(1):358-367.
17. Li M, Grewal J, Hinkle SN, Yisahak SF, Grobman WA, Newman RB, et al. Healthy dietary patterns and common pregnancy complications: a prospective and longitudinal study. Am J Clin Nutr. 2021 Sep 1;114(3):1229-1237.
18. Díaz-López A, Díaz-Torres S, Martín-Luján F, Basora J, Arija V. Prenatal adherence to the Mediterranean diet decreases the risk of having a small-for-gestational-age baby, ECLIPSES study. Sci Rep. 2022 Aug 13;12(1):13794.
19. Ashwin D, Gibson L, Hagemann E, D'Vaz N, Bear N, Silva D. The impact a Mediterranean Diet in the third trimester of pregnancy has on neonatal body fat percentage. J Dev Orig Health Dis. 2022 Aug;13(4):500-507.
20. Fernández-Barrés S, Vrijheid M, Manzano-Salgado CB, Valvi D, Martínez D, Iñiguez C, et al. Infancia y Medio Ambiente (INMA) Project. The Association of Mediterranean Diet during Pregnancy with Longitudinal Body Mass Index Trajectories and Cardiometabolic Risk in Early Childhood. J Pediatr. 2019 Mar;206:119-127.e6.
21. Mantzoros CS, Sweeney L, Williams CJ, Oken E, Kelesidis T, Rifas-Shiman SL, et al. Maternal diet and cord blood leptin and adiponectin concentrations at birth. Clin Nutr. 2010 Oct;29(5):622-6.
22. Karamanos B, Thanopoulou A, Anastasiou E, Assaad-Khalil S, Albache N, Bachaoui M, et al. MGSD-GDM Study Group. Relation of the Mediterranean diet with the incidence of gestational diabetes. Eur J Clin Nutr. 2014 Jan;68(1):8-13.
23. Bédard A, Northstone K, Henderson AJ, Shaheen SO. Mediterranean diet during pregnancy and childhood respiratory and atopic outcomes: birth cohort study. Eur Respir J. 2020 Mar 12;55(3):1901215.
